# Aligned Fingolimod-Releasing Electrospun Fibers Increase Dorsal Root Ganglia Neurite Extension and Decrease Schwann Cell Expression of Promyelinating Factors

**DOI:** 10.3389/fbioe.2020.00937

**Published:** 2020-08-14

**Authors:** Devan L. Puhl, Jessica L. Funnell, Anthony R. D’Amato, Jonathan Bao, Dmitri V. Zagorevski, Yelena Pressman, Daniel Morone, Agnes E. Haggerty, Martin Oudega, Ryan J. Gilbert

**Affiliations:** ^1^Department of Biomedical Engineering, Rensselaer Polytechnic Institute, Troy, NY, United States; ^2^Center for Biotechnology and Interdisciplinary Studies, Rensselaer Polytechnic Institute, Troy, NY, United States; ^3^Department of Biological Sciences, Rensselaer Polytechnic Institute, Troy, NY, United States; ^4^The Miami Project to Cure Paralysis, University of Miami Miller School of Medicine, Miami, FL, United States; ^5^Shirley Ryan AbilityLab, Chicago, IL, United States; ^6^Department of Physical Therapy and Human Movement Sciences and Department of Physiology, Northwestern University, Chicago, IL, United States; ^7^Affiliated Cancer Hospital, Guangzhou Medical University, Guangzhou, China; ^8^Edward Hines, Jr. VA Hospital, Hines, IL, United States

**Keywords:** biomaterial, electrospun fibers, drug delivery, fingolimod hydrochloride, peripheral nervous system injury, dorsal root ganglia, neurons, Schwann cells

## Abstract

Researchers are investigating the use of biomaterials with aligned guidance cues, like those provided by aligned electrospun fibers, to facilitate axonal growth across critical-length peripheral nerve defects. To enhance the regenerative outcomes further, these aligned fibers can be designed to provide local, sustained release of therapeutics. The drug fingolimod improved peripheral nerve regeneration in preclinical rodent models by stimulating a pro-regenerative Schwann cell phenotype and axonal growth. However, the systemic delivery of fingolimod for nerve repair can lead to adverse effects, so it is necessary to develop a means of providing sustained delivery of fingolimod local to the injury. Here we created aligned fingolimod-releasing electrospun fibers that provide directional guidance cues in combination with the local, sustained release of fingolimod to enhance neurite outgrowth and stimulate a pro-regenerative Schwann cell phenotype. Electrospun fiber scaffolds were created by blending fingolimod into poly(lactic-co-glycolic acid) (PLGA) at a w/w% (drug/polymer) of 0.0004, 0.02, or 0.04%. We examined the effectiveness of these scaffolds to stimulate neurite extension *in vitro* by measuring neurite outgrowth from whole and dissociated dorsal root ganglia (DRG). Subsequently, we characterized Schwann cell migration and gene expression *in vitro*. The results show that drug-loaded PLGA fibers released fingolimod for 28 days, which is the longest reported release of fingolimod from electrospun fibers. Furthermore, the 0.02% fingolimod-loaded fibers enhanced neurite outgrowth from whole and dissociated DRG neurons, increased Schwann cell migration, and reduced the Schwann cell expression of promyelinating factors. The *in vitro* findings show the potential of the aligned fingolimod-releasing electrospun fibers to enhance peripheral nerve regeneration and serve as a basis for future *in vivo* studies.

## Introduction

Peripheral nervous system (PNS) injury due to disease or trauma leads to sensory and/or motor dysfunction, which significantly reduces the patient’s quality of life. In the United States, over 20 million people live with symptoms associated with PNS injury (National Institute of Neurological Disorders and Stroke, 2019). Annually, approximately 560,000 peripheral nerve surgeries are performed, totaling 1.68 billion dollars in medical expenses per year (Brattain, 2014). The transected peripheral nerves regenerate readily over short distances, but gaps between the proximal and the distal nerve stumps greater than 1–2 cm in length typically require surgical intervention where a construct is placed to bridge the gap and facilitate axonal regeneration. An autograft is the current gold standard for peripheral nerve reconstruction, but with larger injury gaps, the availability of an autograft with appropriate length and diameter is limited and has the potential to cause donor site morbidity (Deumens et al., 2010). Decellularized allografts are a viable alternative to autografts. Allografts are comprised of native extracellular matrix that supports and directs axonal regeneration, but the availability of cadaveric tissues is limited and the decellularization and the sterilization processes are labor-intensive (Fishman et al., 2012; Safa et al., 2019).

To overcome the limitations of autografts and allografts, artificial nerve grafts have been developed and tested (Arslantunali et al., 2014; López-Cebral et al., 2017; Houshyar et al., 2019; Kornfeld et al., 2019; Qian et al., 2018, 2019a, b). Currently, there are nine commercially available biodegradable artificial nerve grafts for peripheral nerve reconstruction; however, these products are only approved for gap distances of 3 cm or less (Kornfeld et al., 2019). For larger injury gaps, the efficacy of artificial nerve grafts requires improvement. Several research groups are developing artificial nerve grafts that contain features that mimic the aligned organization of the native nervous system extracellular matrix (Arslantunali et al., 2014; Quan et al., 2016; Qian et al., 2018, 2019a, b; Houshyar et al., 2019). Electrospinning can be used to generate a polymer scaffold with highly aligned topography. Aligned electrospun fibers increase neurite density and length compared with outgrowth as observed on flat polymer surfaces *in vitro* (Hurtado et al., 2011). In preclinical rat peripheral nerve injury models, aligned electrospun fibers guide axonal growth across critical gap distances by providing directed contact guidance (Kim et al., 2009; Zhu et al., 2011; Jiang et al., 2014; Xie et al., 2014; Quan et al., 2019). To further increase axonal regeneration, electrospun fibers may be functionalized or designed to release bioactive molecules (Xie et al., 2014; Johnson et al., 2016; Xia and Lv, 2018; Kong et al., 2019).

One small-molecule drug that is currently being investigated for various neural regeneration applications is fingolimod hydrochloride. Fingolimod (marketed as Gilenya^®^) is a Food and Drug Administration-approved immunosuppressant drug used to treat relapsing forms of multiple sclerosis (MS) (Pelletier, 2012). Fingolimod is a sphingosine-1 analog that is activated upon phosphorylation *in vivo* or *in vitro* (Paugh et al., 2003) and binds to four of the five known G-protein coupled sphingosine-1-phosphate (S1P) receptors: S1P_1_, S1P_3_, S1P_4_, and S1P_5_ (Brinkmann et al., 2002). These receptors are ubiquitously expressed by cells in disparate physiological systems (Blaho and Hla, 2014). In the nervous system, Schwann cells and dorsal root ganglia (DRG) neurons express all four of the fingolimod-binding S1P receptor subtypes (Kays et al., 2012; Köhne et al., 2012). Several studies showed that fingolimod-mediated modulation of S1P signaling promoted the conversion of Schwann cells to a regenerative phenotype and increased neurite outgrowth as well as neuronal production of brain-derived neurotrophic factor (BDNF) (Deogracias et al., 2012; Doi et al., 2013; Heinen et al., 2015; Anastasiadou and Knöll, 2016). Additionally, in a preclinical mouse peripheral nerve injury model, a daily intraperitoneal injection of fingolimod promoted peripheral nerve regeneration *via* modulation of S1P signaling (Szepanowski et al., 2016).

**FIGURE 1:**
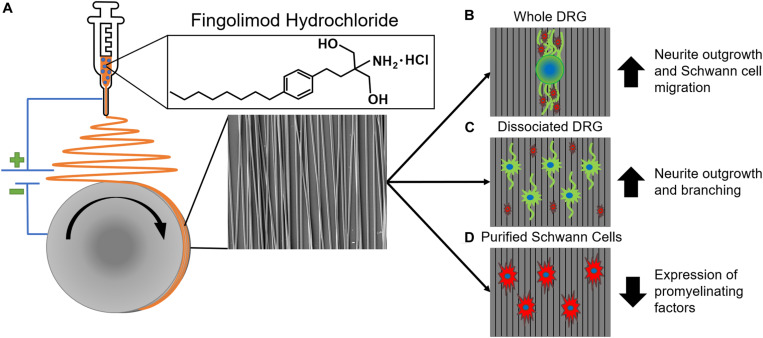
An overview of aligned fingolimod-releasing electrospun fiber fabrication and *in vitro* assessment. **(A)** Fingolimod hydrochloride was incorporated into aligned poly(lactic-co-glycolic acid) fibers *via* blend electrospinning. Aligned fingolimod-releasing fibers **(B)** increased whole dorsal root ganglia (DRG) neurite (green) extension and Schwann cell (red) migration, **(C)** increased individual DRG neuron (green) neurite outgrowth in dissociated DRG cultures, and **(D)** decreased Schwann cell (red) expression levels of promyelinating factors in purified Schwann cell cultures.

Fingolimod is typically taken *vi*a oral administration for the treatment of MS, and since S1P receptors are expressed throughout the body, oral administration can lead to adverse side effects (Blaho and Hla, 2014). For example, patients who take fingolimod orally for nervous system treatment may experience reduced lymphocyte counts, which can lead to an increased chance of infection (Kaufmann et al., 2018). Thus, it would be beneficial to develop biomaterial constructs that provide local, sustained delivery of fingolimod to target a peripheral nerve injury while limiting systemic side effects. In this study, 0.0004, 0.02, or 0.04% (w/w) fingolimod was incorporated into highly aligned poly(lactic-co-glycolic acid) (PLGA) electrospun fibers *via* blend electrospinning (Figure 1A), and fingolimod release was characterized *via* liquid chromatography-mass spectrometry (LCMS). Next, we cultured whole and dissociated DRG on the aligned fibers and assessed changes in neurite outgrowth (Figures 1B,C). Finally, we investigated the ability of fingolimod-releasing fibers to shift Schwann cell phenotype by assessing Schwann cell migration from whole DRG (Figure 1B) and changes in Schwann cell gene expression *via* quantitative polymerase chain reaction (qPCR) (Figure 1D). To our knowledge, this is the first study to show the effect of highly aligned fingolimod-releasing electrospun fibers on Schwann cell phenotype and neurite extension from whole and dissociated DRG neurons.

## Materials and Methods

### Materials

All product information on materials and equipment is listed in Supplementary Tables S1, S2.

### PLGA Film Casting Onto Glass Coverslips

PLGA (lactic acid/glycolic acid ratio—50:50) films were drop-cast onto 15 × 15-mm Ted Pella glass coverslips before electrospinning to help secure the fibers to the surface of the coverslips throughout the cell culture process and to ensure that the cells contact a single material type. A 4% w/w solution of PLGA in 1,1,1,3,3,3-hexafluoro-2-propanol (HFP) was mixed on a stir plate for 1 h at room temperature. A total of 50 μl of PLGA solution was drop-cast onto each glass coverslip. The films were placed in a vacuum chamber overnight to remove any residual HFP before electrospinning.

### Fabrication of Electrospun Fibers

A 1-mg/ml stock of fingolimod was created by completely dissolving fingolimod in the highly versatile solvent HFP (Colomer et al., 2017). A 12% w/w PLGA electrospinning solution was made by dissolving 240 mg of PLGA into 2 g of HFP. Different amounts of fingolimod hydrochloride were blended into the electrospinning solution (bringing the final mass of the solvent HFP to 2 g) for 1 h on a stir plate to fabricate the electrospun fiber types described in Table 1. The loading concentrations were selected in an attempt to obtain release at an effective dose while maintaining consistent fiber morphology.

**TABLE 1:** Fiber groups fabricated for the study.

Mass of fingolimod added to electrospinning solutions (μg)	w/w% of fingolimod to poly(lactic-co-glycolic acid)
0	0.0% (control)
1	0.0004%
50	0.02%
100	0.04%

The electrospinning setup used here is described in detail in a previously published manuscript (Wang et al., 2009). In brief, double-sided tape was used to secure the PLGA film-coated glass coverslips to a spinning disk (22 cm diameter, 1 cm thick). The electrospinning solution was placed into a 5-ml syringe with a 22-G needle and electrospun using the following electrospinning parameters: 10 min—collection time, 4 cm—collection distance, 15 kV—applied voltage, 2 ml/h—solution flow rate, 1,000 rpm—wheel rotation speed, and 21 ± 1%—relative humidity. The electrospun fiber scaffolds were sterilized for 12 h *via* ethylene oxide sterilization and degassed for at least 3 days in a sterile cell culture hood. Three separate solutions were prepared and electrospun per fiber group to fabricate materials in triplicate (*n* = 3).

### Scanning Electron Microscopy and Fiber Morphological Characterization

Electrospun fiber scaffolds were sputter-coated with gold/palladium using a Technics Hummer V Sputter Coater and imaged *via* scanning electron microscopy (SEM) using an FEI Versa 3D Dual Beam SEM. The following imaging parameters were used: 5.0 kV—accelerating voltage, 10 mm—working distance, 5.0 nm—spot size, 30 μm—aperture, and ×2,500—magnification.

Fiber alignment, diameter, and percent coverage were characterized using FIJI Software as described previously (D’Amato et al., 2019b) to ensure consistency between each fiber group and isolate fingolimod release as the sole variable. A total of 300 fiber angles (100 fiber angles per replicate) and 300 fiber diameters (100 fiber diameters per replicate) were measured for each fiber type, and at least three fields of view were assessed for percent fiber coverage. For each fiber type, at least one electrospun fiber scaffold per replicate was imaged *via* SEM and characterized *via* FIJI Software to obtain fiber alignment, diameter, and percent coverage analyses in triplicate (*n* = 3).

### Electrospun Fiber Surface Wettability Assessment

Surface wettability was assessed for each fiber type using a Kruss DSA 100 goniometer as described previously (Ziemba et al., 2020) to determine if the inclusion of fingolimod into the electrospun fibers changed the fiber surface chemistry. At least three drops were assessed per replicate. For each fiber group, the static water contact angle was assessed on at least one electrospun fiber scaffold per replicate to obtain data in triplicate (*n* = 3).

### Fingolimod Release Quantification

#### Sample Collection and Preparation

A large quantity of electrospun fibers was needed to accurately quantify fingolimod release, as fingolimod released from a single fingolimod-loaded electrospun fiber scaffold was below the limit of quantification for all fiber groups. Each fiber group was electrospun directly onto the collection wheel using the previously stated parameters. Subsequently, the electrospun fibers were removed from the wheel and placed under vacuum for at least 3 days to remove any retained solvent. Then, 350 mg of each fiber type was completely submerged in 5 ml of deionized water in an airtight vial and placed in a 37°C cell culture incubator. The entire 5 ml of deionized water was collected and replaced with 5 ml of fresh deionized water on days 1, 2, 3, 4, 7, 10, 14, 21, and 28. After collection, the samples were stored in a −20°C freezer until analysis. Three separate 350-mg bundles of fibers were fabricated per fiber type from separate electrospinning solutions to collect drug release in triplicate (*n* = 3).

Before quantifying fingolimod release, all samples were lyophilized and resuspended in varying volumes of deionized water, ranging from 30 to 100 μl, to bring the fingolimod release samples to a quantifiable concentration.

#### Fingolimod Release Characterization *via* LCMS

The concentration of fingolimod from each 350-mg fiber release sample was measured *via* LCMS. First, a stock solution of fingolimod at a concentration of 20 μM was prepared in deionized water. This solution was further diluted in deionized water to create a standard curve ranging from 50 nM to 20 μM. Standard and sample solutions were pipetted into polypropylene inserts within autosampler vials. Isotopically labeled fingolimod-D4 hydrochloride was used as the internal reference standard. Fingolimod-D4 was brought to a concentration of 10 μM in deionized water. It was then added at a 1:10 dilution to each standard and sample solution, leading to a final concentration of 1 μM of fingolimod-D4 hydrochloride in each solution. The method used for quantifying fingolimod released from fibers was modified from Suneetha and Raja Rajeswari and adjusted to the available instrumentation and column (Suneetha and Raja Rajeswari, 2016). Briefly, an injection volume of 8 μl was fed to an Agilent 1200 HPLC system for separation, operated using Agilent LC OpenLAB Software. Fingolimod and fingolimod-D4 were eluted from a ZORBAX StableBond C18 column (50 × 2.1 mm, particle size of 5 μm, and pore size of 80 Å) using a gradient flow of 0.2% formic acid in water (solvent A) and 0.2% formic acid in acetonitrile (solvent B) as described in Table 2. Standard and sample solutions were eluted from the column at a constant flow rate of 300 μl/min, with a total run time of 15 min. A TSQ Quantum Ultra^TM^ Triple Quadrupole mass spectrometer was operated in electrospray ionization positive ion mode, and Xcalibur^®^ data system was used to acquire and process the data. Selected reaction monitoring was used for the detection of fingolimod and fingolimod-D4: *m/z* 308.3 to *m/z* 255.20 transition (collision energy, 20 V) was used for fingolimod and *m/z* 312.3 to *m/z* 257.50 transition (collision energy, 12 V) was used for fingolimod-D4. The resulting chromatograms were used to calculate the concentration of fingolimod in each 350-mg fiber release sample. Fingolimod release was quantified in triplicate (*n* = 3).

**TABLE 2:** Gradient elution scheme for high-performance liquid chromatography.

Time (min)	% A	% B
0	95	5
8	35	65
10	10	90
10.2	95	5
15	95	5

#### Plotting Fingolimod Release

The cumulative release (in ng) from the 350-mg fiber samples was plotted on a scatter plot. Next, the mass of fibers collected on an individual coverslip for each fiber group was determined by weighing the individual electrospun fiber scaffolds from at least three separate spins (*n* = 3). The mass of the electrospun fibers on an individual electrospun fiber scaffold was determined to be 3 mg for each drug-loaded fiber group; thus, the 350-mg bundle of fibers used to quantify cumulative release was equivalent to 116.67 individual fingolimod-loaded fiber scaffolds. The factor of change between the mass of fibers used to determine cumulative release for each group (350 mg) and the mass of fibers per individual scaffold for each group (3 mg) was used to calculate the predicted cumulative fingolimod release from an individual fiber scaffold for each drug-loaded fiber group. The predicted cumulative release from 3 mg of fibers was plotted on a scatter plot.

To better understand the dissolution of fingolimod from the PLGA fibers, we assessed how well several widely used mathematical models fit the cumulative fingolimod release data, including the zero-order model, first-order model, Higuchi model, Korsmeyer–Peppas model, and Hixson–Crowell’s cube root model (Higuchi, 1963; Ritger and Peppas, 1987; Costa and Sousa Lobo, 2001; Siepmann and Peppas, 2001; Dash et al., 2010; de Mello and Ricci-Júnior, 2011; da Costa et al., 2015; Moydeen et al., 2018; Paolino et al., 2019; Pourtalebi Jahromi et al., 2020; Wu et al., 2020). The mathematical equation and axes of the plot for each model are listed in Table 3. The analysis was only conducted using the cumulative release data from 350 mg of fibers, as the predicted cumulative release data from 3 mg of fibers would yield the same results. The theoretical cumulative amount of fingolimod loaded in each fiber type, which is equivalent to the theoretical cumulative amount of fingolimod released at time infinity (CR∞), was calculated based on the w/w% of fingolimod/PLGA in each fiber group (0.0004, 0.02, and 0.04%) and the mass of the fibers used to collect the release samples (350 mg). The CR∞ was determined to be 1.458 μg for the 0.0004% fibers, 72.917 μg for the 0.02% fibers, and 145.833 μg for the 0.04% fibers. The goodness of fit was determined by the resulting *R*^2^ values. Furthermore, the release exponent (*n*) of the Korsmeyer–Peppas model was closely evaluated for each fiber group, as this value indicates the mechanism of transport of the drug through a polymer matrix. For cylindrical systems like the aligned PLGA fibers, *n* = 0.45 indicates Fickian diffusion, 0.45 < *n* < 0.89 indicates anomalous transport, and *n* = 0.89 indicates case II transport (zero-order) (Ritger and Peppas, 1987; Siepmann and Peppas, 2001).

**TABLE 3:** Kinetic models used to assess drug release data.

Kinetic model	Equation	Plot
Zero-order	CR*t*/CR∞ = *K*_0_*t*	CR*t*/CR∞ *vs.* time
First-order	ln(1- CR*t*/CR∞) = -*K*_1_*t*	ln(1- CR*t*/CR∞) *vs.* time
Higuchi	CR*t*/CR∞ = *K*_H_√*t*	CR*t*/CR∞ *vs.* √(time)
Korsmeyer–Peppas	ln(CR*t*/CR∞) = *n* ln(*t*) + ln(*K*_KP_)	ln(CR*t*/CR∞) *vs.* ln(time)
Hixson–Crowell	1 − (1 − CR*t*/CR∞)^∧^(1/3) = −*K*_HC_*t*	1 − (1 − CR*t*/CR∞)^∧^(1/3) *vs.* time

*t, time (days); CRt, cumulative release at time t; CR∞, theoretical cumulative drug released at time infinity; K_0_, zero-order release constant; K_1_, first-order release constant; K_H_, Higuchi dissolution constant; K_KP_, Korsmeyer–Peppas release constant; n, Korsmeyer–Peppas release exponent; K_HC_, Hixson–Crowell release constant.*

### PLGA Fiber Degradation

*In vitro* PLGA electrospun fiber degradation was visualized over time to determine whether these aligned electrospun fiber scaffolds maintain their guidance cues for an extended period and to investigate possible mechanisms for how fingolimod is released from the fibers. To assess PLGA fiber degradation, control PLGA fibers were completely submerged in 1 ml of serum-containing media and placed in a 37°C cell culture incubator. Each week for 6 weeks (days 7, 14, 21, 28, 35, and 42), a scaffold was removed from the media, washed three times with deionized water to remove any salt or protein debris, and dried on the benchtop. Then, each fiber scaffold was imaged *via* SEM using previously described procedures. The control PLGA fibers were fabricated from separate electrospinning solutions to visualize fiber degradation in triplicate (*n* = 3).

### Whole DRG Isolation, Culture, and Percent Adhesion Analysis

The following procedures were approved by the Institutional Animal Care and Use Committee (IACUC) at Rensselaer Polytechnic Institute. Whole DRG were isolated from 2-day-old Sprague Dawley rats (P2) as described previously (D’Amato et al., 2019b).

Before culturing the whole DRG, all fiber types were plasma-treated on high for 1 min using an expanded plasma cleaner to improve cell adhesion. Next, 500 μl of neuron media, containing neurobasal medium supplemented with 50 ng/ml nerve growth factor, 1% penicillin-streptomycin, 1% B-27, and 0.5 mM L-glutamine, was placed in each well. Two DRG were carefully placed on each scaffold in a manner so that their extending neurites would not interfere with one another. The explants were then cultured at 5% CO_2_ and 37°C in a cell culture incubator. After 24 h, another 500 μl of neuron media was added to each well to bring the total volume of media up to 1 ml. Whole DRG were returned to the incubator and cultured for a total of 4 days. This time-point was selected to allow significant neurite outgrowth while preventing the neurites from growing off the edge of the coverslip. DRG were acquired from at least three separate animals to achieve biological triplicate (*n* = 3).

The total number of whole DRG plated on day 0 and the total number of whole DRG that remained adhered after 4 days in culture were recorded and used to calculate the total percent adhesion of the whole DRG to each fiber type.

### DRG Dissociation and Culture

DRG from two rats were isolated as described previously (D’Amato et al., 2019b) and placed in chilled Ham’s F12 nutrient mixture for dissociation. DRG were centrifuged at 300 rcf for 5 min, and the F12 media mixture was carefully pipetted off. The DRG were then resuspended and incubated at 37°C for 50 min in 2 ml of phosphate-buffered saline (PBS) solution containing 1 mg/ml collagenase and 0.1% trypsin. Throughout the incubation, the DRG were resuspended every 15 min to improve the enzymatic dissociation. Following the 50-min incubation, the cells were centrifuged at 300 rcf for 5 min and the supernatant was carefully pipetted off. The cells were resuspended in 2 ml of PBS solution containing 0.25% (v/v) trypsin and incubated at 37°C for 10 min. Next, 3 ml of serum-containing media was added to neutralize the trypsin. The cells were then centrifuged again at 300 rcf for 5 min, the supernatant was carefully removed, and the cells were resuspended in neuron media. Three separate dissociations were performed and DRG were isolated from six animals to achieve biological triplicate (*n* = 3).

Before plating the cells acquired through DRG dissociation, fiber scaffolds and glass coverslips (used for purity assessment—reported in the Supplementary Material) were placed in a 12-well culture plate and coated with 50 μg/ml laminin in deionized water to promote cell adhesion. After coating for 3 h in a cell culture incubator, the fiber scaffolds and glass coverslips were washed three times with deionized water. Following DRG dissociation, the cells were resuspended in neuron media at a concentration of 30,000 cells/ml. One milliliter of cell suspension (30,000 cells per well) was added to each well, and the cells were cultured for a total of 12 h in a cell culture incubator. This time-point was selected to allow significant neurite outgrowth while preventing the neurites from growing into one another.

### Schwann Cell Isolation, Purification, and Culture

The following procedures were approved by the IACUC at the University of Miami. Purified populations of Schwann cells were obtained from the sciatic nerves of 3-day-old Sprague Dawley rats (P3) as described previously (Brockes et al., 1979). The Schwann cells were purified and expanded as described previously (Kanno et al., 2014). The cells were grown to confluency and passaged to new dishes two times before being cryopreserved until use. The resulting Schwann cell cultures were 95% pure based on S100 immunostaining (Takami et al., 2002).

Before placement onto fiber scaffolds, the Schwann cells were thawed, brought up in the Schwann cell media containing DMEM, 10% fetal bovine serum, 3 μM forskolin, and 1.25 nM heregulin, and cultured for 1 week in a T75 flask with media changes biweekly. After 1 week, the Schwann cells were lifted using TrypLE Express and seeded onto plasma-treated control and 0.02% fingolimod-loaded electrospun fiber scaffolds in a 12-well culture plate. This assessment was conducted on the 0.02% fingolimod-loaded fibers, as this seemed to be the optimal loading concentration according to whole and dissociated DRG neurite extension analysis. At this point, the Schwann cells were passaged a total of three times. The cells were seeded at a density of 300,000 cells per scaffold for the qPCR experiments and to capture high-density fluorescent images and a density of 10,000 cells per scaffold to capture low-density fluorescent images. The Schwann cells were cultured for 4 days before lysing for qPCR or fixation for fluorescent imaging due to preliminary findings (Supplementary Figures S6, S7). Polydimethylsiloxane molds were fabricated to concentrate the Schwann cells directly onto the fibers, preventing the cells from attaching to the well plate that surrounds the scaffold. The Schwann cells were cultured on three separate material replicates to achieve triplicate (*n* = 3).

### Cell Immunocytochemistry and Confocal Imaging

Whole DRG and purified Schwann cells, cultured for 4 days, and dissociated DRG, cultured for 12 h, were fixed for 15 min in a solution of 4% (v/v) paraformaldehyde in PBS. Following fixation, the cells were washed three times with PBS. Whole DRG and purified Schwann cells were blocked for 1 h, and the dissociated DRG cultures were blocked for 15 min in a PBS solution containing 5% (w/v) bovine serum albumin (BSA) and 0.01% (v/v) Triton X-100. The blocking solution was removed and whole DRG, purified Schwann cell, and dissociated DRG cultures were incubated overnight at 4°C in a primary antibody solution containing 5% BSA and 0.1% (v/v) TWEEN-20. The purified Schwann cell primary antibody solution contained a 1:500 dilution of rabbit polyclonal S100 primary antibody in PBS. The whole and dissociated DRG primary antibody solution contained a 1:500 dilution of mouse polyclonal RT-97 primary antibody and a 1:500 dilution of rabbit polyclonal S100 primary antibody in PBS. The RT-97 primary antibody is specific to neurofilament and the S100 primary antibody is commonly used to identify Schwann cells in culture. On the following day, whole DRG, purified Schwann cells, and dissociated DRG cultures were washed twice with PBS and then incubated for 1 h in a secondary antibody solution containing 5% BSA and 0.1% TWEEN-20. The purified Schwann cell secondary antibody solution contained a 1:1,000 dilution of Alexa Fluor goat anti-rabbit 594 secondary antibody in PBS. The whole and dissociated DRG secondary antibody solution contained a 1:1,000 dilution of Alexa Fluor donkey anti-mouse 488 secondary antibody and a 1:1,000 dilution of Alexa Fluor goat anti-rabbit 594 secondary antibody in PBS. After 45 min, a 1:1,000 dilution of 4′,6-diamidino-2-phenylindole (DAPI) nuclear stain was added into each well for the remaining 15 min of incubation. After 1 h, the secondary antibody solution was removed, and all cultures were washed three times in PBS. All stained cultures were stored in 1 ml of PBS at 4°C until imaged.

The cells were imaged using Metamorph Premier 7.7.3.0 imaging software and a 289 Olympus IX-81 confocal microscope and processed as described previously (D’Amato et al., 2019b). Whole DRG, Schwann cells, and dissociated DRG were captured at a magnification of ×4, ×10, and ×20, respectively.

### Neurite Outgrowth Analysis From Whole DRG and Individual DRG Neurons

Neurite outgrowth from whole DRG explants was determined using images of whole DRG cultured on each fiber type and stained for neurofilament to investigate whether fingolimod released from electrospun fibers enhances neurite extension. Using FIJI software, the lengths of the five longest neurites on each side of the DRG body were measured by drawing a line from the edge of the DRG body to the tip of each neurite (D’Amato et al., 2019a). The measurements were recorded and the average neurite length per side was calculated. Neurite outgrowth from each side of the DRG body was considered as an individual replicate. Mean neurite extension was determined by assessing seven to 15 whole DRG per fiber type (*n* = 14–30). The number of whole DRG assessed differs per fiber type due to variations in DRG adhesion.

To further assess whether fingolimod released from electrospun fibers improves neurite outgrowth, individual neuron morphology was investigated using Neurolucida software and images of dissociated DRG cultured onto each fiber type and stained against neurofilament as described previously (D’Amato et al., 2019b). Neurolucida Explorer software was then used to compute the total neurite length, longest neurite length, total number of branch points, and total number of primary neurites per individual neuron. A total of 30–33 individual neurons from a total of three separate dissociations were assessed per fiber type (*n* = 30–33).

### Schwann Cell Migration Assessment

Maximum Schwann cell migration distance from the whole DRG body was measured using images of whole DRG stained with DAPI to determine if fingolimod released from electrospun fibers affects Schwann cell migration. Schwann cell migration from the whole DRG body was assessed as described previously (Wang et al., 2010). To ensure that the DAPI-stained nuclei on either side of the DRG body indicated the presence of Schwann cells, images of the whole DRG stained for S100 and counterstained with DAPI were compared. The images utilized to assess whole DRG neurite extension were also used for this analysis, and each side of the DRG body was considered as an individual replicate. Maximum Schwann cell migration from the whole DRG body was determined by assessing seven to 15 whole DRG images per fiber type (*n* = 14–30).

### Schwann Cell Gene Expression Analysis

qPCR was used to determine if fingolimod released from electrospun fibers affected Schwann cell gene expression. To determine the optimal time-point to assess Schwann cell gene expression, the Schwann cells were exposed to 100 nM fingolimod, a dose shown to effectively shift Schwann cells toward a pro-regenerative phenotype by Heinen et al. (2015), for either 1 or 4 days. We observed the largest changes in Schwann cell gene expression after 4 days. Thus, Schwann cell gene expression was assessed following 4 days of culture on the 0.02% fingolimod-loaded fibers. After 4 days in culture, the Schwann cells were lysed with TRIzol, and RNA was isolated and purified according to the manufacturer’s protocol. RNA was reverse-transcribed into cDNA using qScript cDNA SuperMix. The cDNA was combined with primer stocks and PerfeCTa SYBR Green FastMix ROX and was amplified using a STEPOne Real-Time PCR System. The primer set is listed in Table 4. Primer sequences were referenced from Heinen et al. (2015) and checked using BLAST analysis. The relative gene expression was calculated using the ΔΔCt method, with the data normalized to GAPDH and Schwann cells seeded onto control fibers. The Schwann cells were cultured on three separate material replicates to achieve triplicate for qPCR experiments (*n* = 3).

**TABLE 4:** Primer set for assessment of Schwann cell gene expression.

Gene	Sense	Antisense
**Housekeeping**		
GAPDH	GCCTCCAAGGAGTAAGAAAC	GTCTGG GATGGAATTGTGAG
**Regenerative**		
BDNF	GGTATCAAAAGGCCAACTGA	GCAGCCTTCCTTGGTGTAAC
cJun	GACCTTCTACGACGATGCCC	CCACTCTCGGACTGGAGGAAC
GAP43	CCGGAGGATAAGGCTCATAAGG	TTGTTATGTGTCCACGGAAGCT
NCAM1	AAAGGATGGGGAACCCATAG	TAGGTGATTTTGGGCTTTGC
PDGF-BB	GTTCGGACGGTGCGAATC	GTGTGCTTAAACTTTCGGTGCTT
**Myelinating**		
Cx32	CCTCCGGCATCTGCATTATC	AGGCCCGGATGATGAGGTA
Krox20	TTTTTCCATCTCCGTGCCA	TAGGTGATTTTGGGCTTTGC
MBP	CAATGGACCCGACAGGAAAC	TGGCATCTCCAGCGTGTTC
Oct6	GGCACCCTCTACGGTAATGTGT	TTGAGCAGCGGTTTGAGCT
PMP2	TGCAGAAGTGGGATGGTAAAGA	TCCACTACCATTTTCCCATCCA

### Statistical Analysis

Statistical analysis was performed in Minitab 19. First, a Ryan–Joiner test was conducted to determine if the data were normally distributed, and then equal variance of the data was tested. Fiber diameter data were assessed using Welch’s ANOVA with *post hoc* Games–Howell test and represented as mean ± standard deviation. Percent fiber coverage data were assessed using one-way ANOVA with *post hoc* Dunnett’s test and represented as mean ± standard deviation. Percent fiber alignment data were assessed using Mood’s median test and represented by histograms displaying the percentage of fibers with a deviation from the mean fiber angle in degrees. Static water contact angle data were assessed using Welch’s ANOVA and *post hoc* Games–Howell test compared with the control fiber group and represented as mean ± standard deviation. Percent whole DRG adhesion data were assessed *via* a binary logistic regression and represented as the percentage of whole DRG that remained adhered to the fiber group after 4 days in culture out of the total number of whole DRG initially cultured on day 0. Significant differences in whole DRG neurite extension data compared with the control fiber group were determined using one-way ANOVA and *post hoc* Dunnett’s test and represented as mean ± standard error of the mean. Statistical differences in individual neuron total neurite length, longest neurite length, and number of branch points data compared with the control fiber group were determined *via* Welch’s ANOVA and *post hoc* Games–Howell test. Differences in individual neuron number of primary neurites data compared with the control fiber group were determined using one-way ANOVA and *post hoc* Dunnett’s test. All individual neuron data were displayed as mean ± standard error of the mean. Statistical differences in Schwann cell migration data compared with the control fiber group were assessed *via* one-way ANOVA and *post hoc* Dunnett’s test and are represented as mean ± standard error of the mean. Finally, statistical differences in Schwann cell qPCR data compared with the control fiber group were determined using a general linear regression and represented as mean ± standard error of the mean.

## Results

In this study, we blended various amounts of fingolimod into PLGA solutions and electrospun onto PLGA film-coated coverslips. The control PLGA fibers contained no fingolimod and are designated as 0.0% in all figures. The fingolimod-loaded PLGA fiber scaffolds contained 0.0004, 0.02, or 0.04% w/w fingolimod/PLGA and are designated as such in all figures.

### Morphological Characterization of Electrospun Fiber Scaffolds

**FIGURE 2:**
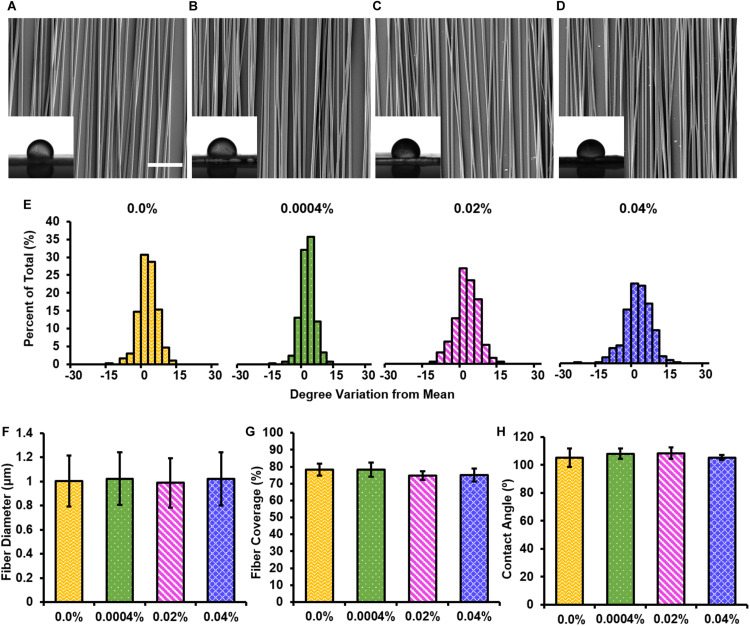
Electrospun fibers containing various loading concentrations of fingolimod share similar morphological features. SEM images of **(A)** 0.0%, **(B)** 0.0004%, **(C)** 0.02%, or **(D)** 0.04% w/w fingolimod/poly(lactic-co-glycolic acid) electrospun fibers with inlaid images of water droplets used to measure static water contact angle for each (scale bar = 20 μm). **(E)** Fiber alignment data for each loading concentration are represented by histograms displaying the percentage of fibers with a deviation from the mean fiber angle in degrees. **(F)** Fiber diameter data are represented as mean diameter (in μm) ± standard deviation. **(G)** Fiber surface coverage data are represented by the mean percentage of the coverslip covered by fibers ± standard deviation. **(H)** Static water contact angle data (in degrees) are represented by the mean contact angle ± standard deviation.

The inclusion of a drug into electrospun fibers may affect their morphological characteristics (Johnson et al., 2016). Using SEM, we investigated the degree of fiber alignment, fiber diameter, and percent fiber coverage on the coverslip of each electrospun fiber group (Figures 2A–D). Note that we studied 0.0004, 0.02, and 0.04% fingolimod-loaded fibers because, in pilot studies, we found that increasing the loading concentration to 0.4% fingolimod resulted in poor fiber formation, inconsistent fiber diameter, and decreased fiber alignment (Supplementary Figure S1). Between our studied groups, fiber alignment did not significantly change when fingolimod concentration was increased (Figure 2E and Supplementary Tables S3, S4). The control fibers had a mean fiber diameter of 1.00 ± 0.21 μm and a mean percent fiber coverage of 78.18 ± 3.47%. The inclusion of fingolimod into the electrospun fibers did not significantly alter the mean fiber diameter or the percent fiber coverage for the 0.0004% (1.02 ± 0.22 μm; 78.26 ± 4.09%), 0.02% (0.988 ± 0.20 μm; 74.71 ± 2.66%), and 0.04% (1.02 ± 0.22 μm; 75.10 ± 3.94%) fiber groups compared with that of the control fibers (Figures 2F,G and Supplementary Tables S5–S8).

### Electrospun Fiber Scaffold Surface Wettability

The inclusion of fingolimod can change fiber surface chemistry and, thus, the hydrophilicity, which may influence cell adhesion and neurite extension (Schaub et al., 2015). The surface wettability of the electrospun fiber groups was measured via the static water contact angle (Figures 2A–D,H). The control fibers had a mean static water contact angle of 105.02 ± 6.58° (Figure 2H). The static water contact angle of the 0.0004% (107.90 ± 3.68°), 0.02% (108.32 ± 4.05°), and 0.04% (105.36 ± 1.67°) fingolimod-loaded fiber groups was not significantly different from that of the control fibers (Figure 2H and Supplementary Tables S9–S11). These results indicate that the inclusion of fingolimod did not alter electrospun fiber surface water interfacial properties.

### Fingolimod Release Kinetics

**FIGURE 3:**
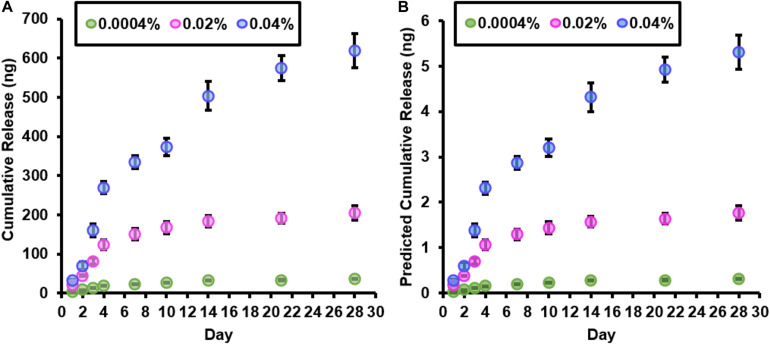
Fingolimod-loaded fibers continuously release fingolimod for at least 28 days. **(A)** Cumulative release (in ng) of fingolimod from 350 mg of 0.0004% (green), 0.02% (pink), and 0.04% (blue) fingolimod-loaded electrospun fibers over 28 days. **(B)** Predicted cumulative release of fingolimod (in ng) from a single 0.0004% (green), 0.02% (pink), and 0.04% (blue) fingolimod-loaded electrospun fiber scaffold over 28 days. An individual fingolimod-loaded electrospun fiber scaffold contains 3 mg of fibers. Data are represented by the mean mass of drug released (in ng) ± standard deviation.

The cumulative release of fingolimod from a 350-mg bundle of electrospun fibers and the predicted cumulative release of fingolimod from a single 3-mg electrospun fiber scaffold for each fingolimod-loaded fiber group are displayed in Figures 3A,B, respectively. Over the first 4 days, a linear release of fingolimod from the 0.0004% (*R*^2^ = 1.0), 0.02% (*R*^2^ = 0.99), and 0.04% (*R*^2^ = 0.96) fiber groups was observed. Thereafter, fingolimod release began to decrease. Following 28 days, the cumulative release of fingolimod predicted for an individual 0.0004, 0.02, and 0.04% fiber scaffold was 0.31 ± 0.01, 1.76 ± 0.16, and 5.31 ± 0.37 ng, respectively (Figure 3B). Separate predicted cumulative release curves are presented in the Supplementary Material to display the release profiles from each fingolimod-loaded fiber group (Supplementary Figures S2A–C). Additionally, graphs displaying the predicted release concentration of fingolimod from a single electrospun fiber scaffold into 1 ml of culture media are provided in Supplementary Figures S2D–G. Although fingolimod release begins to slow down following 4 days, Supplementary Figures S2D–G show that there is a greater than threefold increase in predicted concentration between days 10 and 14 from the 0.04% fibers (0.98 ± 0.17 to 3.23 ± 0.80 nM) and an approximate twofold increase in the predicted concentration between days 21 and 28 from the 0.0004% (0.35 ± 0.06 to 0.070 ± 0.06 nM) and 0.02% (0.19 ± 0.02 to 0.36 ± 0.17 nM) fibers.

To better understand the mechanisms of fingolimod release from the aligned PLGA fibers, several kinetics models were fit to the drug release data (Table 5 and Supplementary Figures S3A–E). The Higuchi model was the best fit for the 0.0004% (*R*^2^ = 0.931) and 0.04% (*R*^2^ = 0.960) fingolimod-releasing fiber groups. The Korsmeyer–Peppas model was the best fit for the 0.02% (*R*^2^ = 0.860) fingolimod-releasing fiber group, and this was closely followed by the Higuchi model (*R*^2^ = 0.858). The Korsmeyer–Peppas release exponent (*n*) values for the 0.0004% (*n* = 0.617), 0.02% (*n* = 0.649), and 0.04% (*n* = 0.865) fiber groups were all within the range of 0.45 < *n* < 0.89, indicating anomalous transport. Plots for each kinetic model are presented in Supplementary Figures S3A–E.

**TABLE 5:** The *R*^2^ and release exponent (*n*) obtained by fitting the kinetic models to the release data.

	Kinetic model					

Fiber group	Zero-order	First-order	Higuchi	Korsmeyer–Peppas		Hixson–Crowell
	*R*^2^	*R*^2^	*R*^2^	*R*^2^	*N*	*R*^2^
0.0004%	0.810	0.812	0.931	0.923	0.617	0.811
0.02%	0.714	0.714	0.858	0.860	0.649	0.714
0.04%	0.870	0.870	0.960	0.909	0.865	0.870

### *In vitro* PLGA Fiber Degradation

**FIGURE 4:**
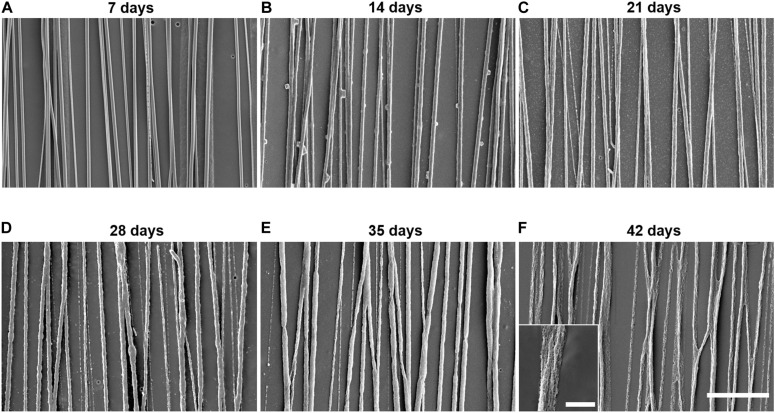
Control poly(lactic-co-glycolic acid) (PLGA) electrospun fibers show significant degradation over 6 weeks. SEM images of control PLGA fibers incubated in cell culture media at 37°C for **(A)** 7, **(B)** 14, **(C)** 21, **(D)** 28, **(E)** 35, or **(F)** 42 days illustrate the gradual degradation of the fiber scaffolds over 6 weeks. The scale bar of these images represents 20 μm. The scale bar of the inlaid image represents 4 μm.

We visualized *in vitro* degradation using SEM images of electrospun PLGA fibers captured throughout the 42-day incubation period (Figure 4). The PLGA fibers do not show signs of significant degradation on day 7 (Figure 4A). Swelling of the PLGA fibers begins between day 7 and day 14 (Figure 4B) of incubation and becomes more apparent between day 21 and day 28 (Figures 4C,D). Furthermore, degradation and pitting occur around day 21 and become more apparent by day 28 (Figures 4C,D). Pitting increases at day 35 (Figure 4E), and significant erosion can be observed by day 42 (Figure 4F). Throughout the entirety of the 42-day incubation, the electrospun PLGA fibers maintain their aligned orientation.

### Whole DRG Adhesion and Neurite Outgrowth

**FIGURE 5:**
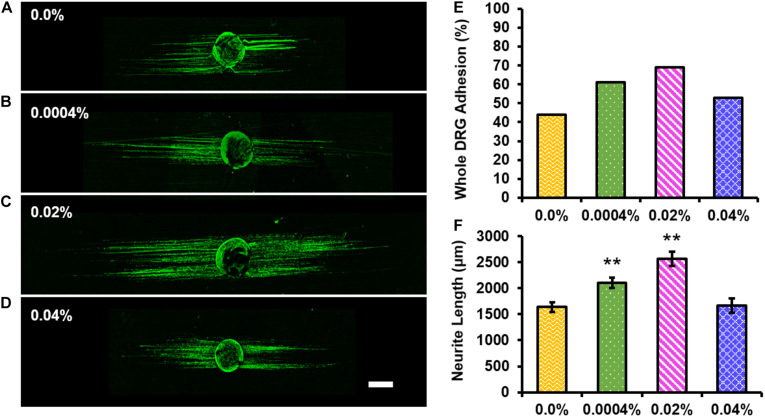
The 0.0004 and 0.02% fingolimod-loaded electrospun fiber groups significantly increased neurite extension from whole dorsal root ganglia (DRG) explants. Confocal images of whole DRG stained against neurofilament (green) and cultured for 4 days on **(A)** 0.0%, **(B)** 0.0004%, **(C)** 0.02%, or **(D)** 0.04% fingolimod-loaded poly(lactic-co-glycolic acid) electrospun fibers (scale bar = 500 μm). **(E)** Whole DRG total percent adhesion data show the percentage of the total number of whole DRG that remained adhered to each fiber type after 4 days in culture. **(F)** Neurite length data are represented by the mean length (in μm) ± standard error of the mean. Statistical significance compared with the 0.0% control fibers was assessed using one-way ANOVA and *post hoc* Dunnett’s test (**p < 0.001).

Representative images of RT-97-stained whole DRG cultured on control (Figure 5A) or 0.0004% (Figure 5B), 0.02% (Figure 5C), or 0.04% (Figure 5D) fingolimod-containing fibers are presented. First, the total percent of DRG that remained adhered to the fiber surface following 4 days in culture was assessed (Figure 5E) to determine if the inclusion of fingolimod into the electrospun fibers affects the ability of whole DRG to adhere to the fiber scaffolds; 44% of whole DRG remained adhered to the drug-free control fibers following the 4-day culture. The percentage of adherent DRG increased to 61 and 69% for the 0.0004 and 0.02% fingolimod-loaded fiber groups and then declined to 53% for the 0.04% fingolimod-loaded fiber group. The data show that, although there is a trend toward increased adhesion with the incorporation of fingolimod into electrospun fibers, this increase is not statistically significant when compared to the drug-free control fibers (see Supplementary Tables S12, S13). Next, we assessed neurite outgrowth from whole DRG explants cultured on the control and fingolimod-releasing PLGA fibers following 4 days in culture (Figure 5F). Figure 5F shows that whole DRG extended significantly longer neurites when cultured on 0.0004% (2,100.82 ± 98.44 μm, *p* = 0.001) and 0.02% (2,570.42 ± 137.58 μm, *p* < 0.001) fingolimod-loaded fiber scaffolds compared with the neurite outgrowth observed on the control fibers (1,639.47 ± 95.57 μm). Alternatively, DRG cultured on the 0.04% (1,663.88 ± 134.92 μm) fingolimod-containing fibers were unable to extend neurites significantly longer than those cultured on the control fibers (Supplementary Tables S14–S16).

### Individual Neuron Morphological Analysis

**FIGURE 6:**
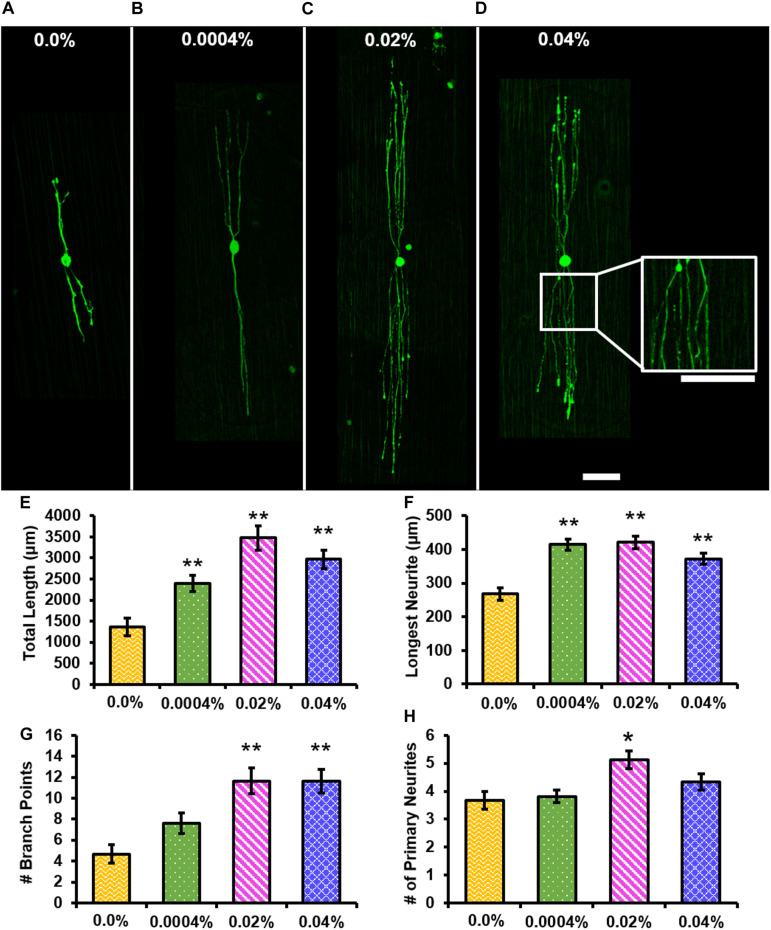
The addition of fingolimod to poly(lactic-co-glycolic acid) (PLGA) electrospun fibers affects neurite outgrowth from individual dorsal root ganglia (DRG) neurons. Confocal images of individual DRG neurons stained against neurofilament (green) and cultured for 12 h on **(A)** 0.0%, **(B)** 0.0004%, **(C)** 0.02%, or **(D)** 0.04% fingolimod-loaded PLGA electrospun fibers (scale bar = 100 μm). **(E)** Total neurite length (in μm), **(F)** longest neurite length (in μm), **(G)** the number of branch points, and **(H)** the number of primary neurites data are represented by the mean ± standard error of the mean. Statistical significance compared with the 0.0% control fibers was assessed for total neurite length, longest neurite length, and the number of branch points data using Welch’s ANOVA and *post hoc* Games–Howell test and for the number of primary neurites using one-way ANOVA and *post hoc* Dunnett’s test (**p < 0.001, *p < 0.05).

Representative images of RT-97-stained individual DRG neurons cultured for 12 h on control (Figure 6A) or 0.0004% (Figure 6B), 0.02% (Figure 6C), or 0.04% (Figure 6D) fingolimod-containing fibers are presented. The individual neurons were assessed after 12 h of culture to prevent the neurites of neighboring neurons from overlapping as neurite extension from individual neurons occurred relatively quickly. Figure 6E compares the total length of neurites extending from the soma of individual neurons and shows that the total neurite length was significantly greater when cultured on 0.0004% (2,393.35 ± 187.73 μm, *p* < 0.001), 0.02% (3,476.43 ± 286.30 μm, *p* < 0.001), and 0.04% (2,965.80 ± 216.42 μm, *p* < 0.001) fingolimod-loaded fibers compared with the total length of neurites observed on control fibers (1,362.15 ± 205.04 μm) (Supplementary Tables S17–S19). Furthermore, Figure 6F presents the length of the longest neurite extending from the soma being significantly longer for neurons cultured on 0.0004% (414.55 ± 17.14 μm, *p* < 0.001), 0.02% (421.00 ± 18.05 μm, *p* < 0.001), and 0.04% (372.46 ± 15.78 μm, *p* < 0.001) fingolimod-loaded fibers compared with the longest neurite length observed on the control fibers (268.08 ± 18.30 μm) (Supplementary Tables S20–S22). Next, the number of neurite branch points and the number of primary neurites extending from the soma were examined (Figures 6G,H). The neurons had significantly more branch points (Figure 6G) when cultured on 0.02% (11.65 ± 1.25 branch points, *p* < 0.001) and 0.04% (11.63 ± 1.13 branch points, *p* < 0.001) fingolimod-loaded fibers compared with the number of branch points observed on control fibers (4.67 ± 0.87 branch points). The individual neurons cultured on the 0.0004% (7.61 ± 1.01 branch points) fingolimod-loaded fibers did not branch appreciably more than those cultured on control fibers (Supplementary Tables S23–S25). The number of primary neurites (Figure 6H) extending from the soma of individual neurons was significantly greater when cultured on 0.02% (5.13 ± 0.32 primary neurites, *p* = 0.002) fingolimod-loaded fibers compared with the number of primary neurites observed on control fibers (3.67 ± 0.31 primary neurites). The individual neurons cultured on 0.0004% (3.82 ± 0.324 primary neurites) and 0.04% (4.34 ± 0.29 primary neurites) fingolimod-loaded fibers, however, did not present a significantly increased number of primary neurites compared with the neurons cultured on control fibers (Supplementary Tables S26–S28). The data indicate that the unique fingolimod release rates observed from these fibers enhance neurite outgrowth from DRG neurons and alter the dynamics of neurite genesis and branching. Because the 0.02% fingolimod-loaded scaffold caused the greatest increase in neurite outgrowth from whole DRG and significantly increased the neurite total length, longest neurite, number of branch points, and number of primary neurites in dissociated DRG neurons, we used this scaffold to assess changes in Schwann cell mRNA expression levels compared to unloaded fibers in subsequent qPCR experiments.

### Schwann Cell Migration From DRG Body

**FIGURE 7:**
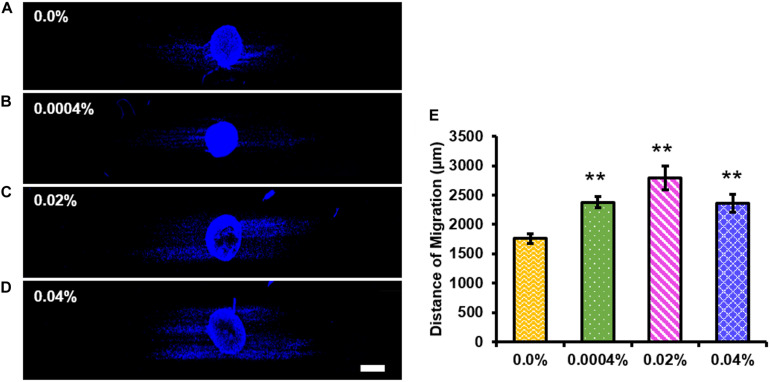
All fingolimod-loaded electrospun fiber groups significantly increased Schwann cell migration outward from the dorsal root ganglia (DRG) body. Confocal images of whole DRG stained with 4′,6-diamidino-2-phenylindole and cultured on **(A)** 0.0%, **(B)** 0.0004%, **(C)** 0.02%, or **(D)** 0.04% fingolimod-loaded poly(lactic-co-glycolic acid) electrospun fibers (scale bar = 500 μm). **(E)** The migration distance data for each fiber group are represented by the mean distance traveled from the DRG body ± the standard error of the mean. Statistical significance compared with the 0.0% control fibers was assessed for Schwann cell migration distance using one-way ANOVA and *post hoc* Dunnett’s test (**p < 0.001).

We assessed the effect of fingolimod-releasing fibers on Schwann cell migration using images of DAPI-stained whole DRG explants (Figures 7A–D). The images of the whole DRG stained against S100 and counterstained with DAPI are displayed in Supplementary Figures S5A–D. Figure 7E shows the quantification of Schwann cell migration from the DRG body and shows that Schwann cells migrated to a greater extent on the 0.0004% (2,379.97 ± 107.86 μm, *p* < 0.001), 0.02% (2,793.03 ± 204.92 μm, *p* < 0.001), and 0.04% (2,359.15 ± 145.48 μm, *p* = 0.001) fingolimod-loaded fibers when compared with the control fibers (1,761.10 ± 80.31 μm) (Supplementary Tables S29–S31).

### Schwann Cell qPCR

**FIGURE 8:**
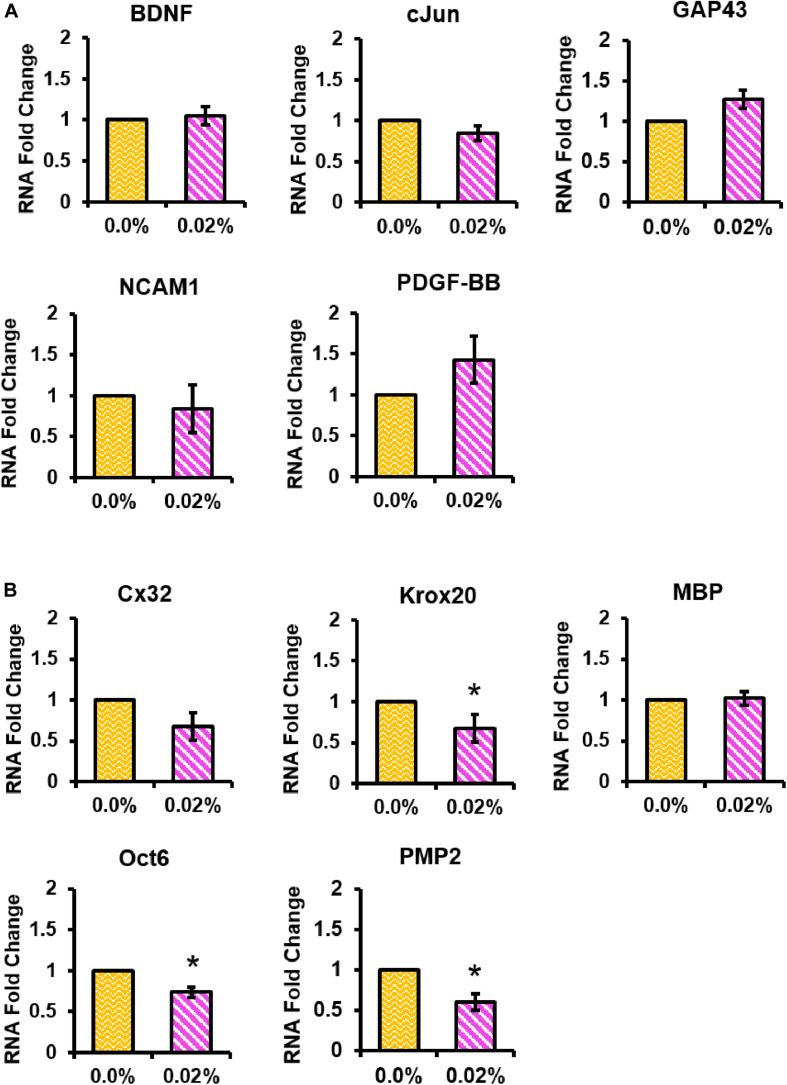
The 0.02% fingolimod-loaded electrospun fibers significantly decreased Schwann cell expression levels of several promyelinating factors. qPCR was conducted to determine Schwann cell mRNA expression levels of the **(A)** regenerative factors BDNF, cJun, GAP43, NCAM1, and PDGF-BB and the **(B)** myelinating factors Cx32, Krox20, MBP, Oct6, and PMP2. Schwann cell gene expression data are represented by the mean RNA fold change ± standard deviation. Statistical significance was assessed using general linear regression (*p < 0.05).

We used qPCR to examine whether fingolimod released from our fibers affected the Schwann cell phenotype. The mRNA expression levels of Schwann cell phenotypic markers were studied in Schwann cells cultured on control or 0.02% fingolimod-loaded fibers (Supplementary Figure S6). A preliminary study revealed that the largest changes in Schwann cell gene expression occurred 4 days after exposure to a 100-nM dose of fingolimod (Supplementary Figures S7, S8). Thus, Schwann cell gene expression was examined following 4 days of culture on the control fibers and 0.02% fingolimod-loaded fibers (Figures 8A,B). The data presented in Figure 8A show that sustained fingolimod release from electrospun fibers did not significantly change the Schwann cell mRNA expression levels of the regenerative factors BDNF, cJun, GAP43, NCAM1, or PDGF-BB (values presented in Supplementary Tables S32–S36). The data presented in Figure 8B show that sustained fingolimod release from electrospun fibers significantly lowered the mRNA levels of the promyelinating factors Krox20 (*T* = 2.86, *p* = 0.046), Oct6 (*T* = 4.49, *p* = 0.011), and PMP2 (*T* = 3.84, *p* = 0.018). Culture on 0.02% fingolimod-loaded fibers, however, did not significantly change the Schwann cell mRNA expression levels of myelinating factors Cx32 and MBP (values presented in Supplementary Tables S37–S41).

## Discussion

The effects of fingolimod-releasing electrospun PLGA fibers on neurite extension and Schwann cells *in vitro* were investigated. We found that fingolimod was continuously released from electrospun fibers for up to 28 days, which significantly increased neurite growth from whole DRG and individual DRG neurons, increased Schwann cell migration from whole DRG, and decreased the Schwann cell expression of promyelinating factors.

The highly aligned topography of electrospun fiber scaffolds is known to facilitate neurite extension *in vitro* and axonal growth *in vivo* compared to randomly orientated electrospun fibers and flat polymer films (Hurtado et al., 2011). Wang et al. (2010) showed that highly aligned poly-L-lactic acid electrospun fibers with a diameter comparable to those used in this study enhanced neurite extension and Schwann cell migration compared with fibers of smaller diameter and reduced alignment. Here, to further enhance their regenerative propensity, we loaded aligned electrospun fibers with fingolimod, which has been associated with improved functional outcomes following peripheral nerve injury (Szepanowski et al., 2016).

Our findings show that fingolimod inclusion did not significantly change fiber alignment, diameter, or percent coverage, which is important because such changes could affect cellular behavior on the fibers (Wang et al., 2010). It is worth noting that, although not significant, the scaffolds became less aligned with increasing fingolimod concentration. Fingolimod contains a polar head group as well as hydrochloride salt, both of which could affect fiber alignment (Song et al., 2011; Johnson et al., 2016). Previously, it was shown that decreased fiber alignment impedes neurite extension and Schwann cell migration (Wang et al., 2010). Thus, our finding that fingolimod inclusion did not significantly alter the fiber alignment supports its use to promote axon growth in the injured peripheral nerve.

Next, fiber surface wettability was examined since changes in fiber surface hydrophilicity can affect cell adhesion and neurite extension (Lee et al., 2003). Our results show that the inclusion of fingolimod into our electrospun PLGA fibers did not change the fiber surface wettability. Amphiphilic molecules such as fingolimod are known to increase the surface hydrophilicity of synthetic electrospun fibers when loaded at high-enough concentrations (Bowers et al., 2017). The findings indicate that we did not load a high-enough concentration of fingolimod to affect the hydrophilicity of the electrospun PLGA fibers; thus, changes in cell behavior are most likely due to the release of fingolimod from the fibers rather than changes in fiber surface chemistry.

The fingolimod release curves and PLGA fiber degradation images help to decipher the mechanism of fingolimod release. With a steady release of fingolimod and no visible fiber swelling or degradation over the first 7 days, we speculate that diffusion is the primary mechanism of release during this time. Although release began to slow down on day 7, successive increases in fingolimod release between days 10 and 14 and between days 21 and 28 coincide with visible fiber swelling, pitting, and erosion. The fiber swelling indicates an increase in the uptake of water by the fibers and therefore results in enhanced diffusion of fingolimod out of the fibers. The physical erosion of fibers during degradation increases the amount of drug that is exposed and released into solution (Chou et al., 2015). Thus, we speculate that both swelling and physical degradation of the fibers led to changes in the drug release kinetics at the later time-points. Additionally, although fingolimod was simply blended into the PLGA fibers, we observed a prolonged release of the drug for at least 28 days. The sustained release may be attributed to fingolimod’s hydrophobic tail. This portion of the molecule likely favored the more hydrophobic environment within the PLGA matrix (Butler et al., 1999; Gentile et al., 2014; Sun et al., 2017; Xu et al., 2017), preventing an initial burst and allowing for a prolonged release of fingolimod (Lao et al., 2009; Xu et al., 2017).

Fitting kinetics models to the release data allows for a more thorough investigation into the mechanisms of fingolimod release from the PLGA fibers. The Higuchi model, which describes drug release from a matrix system, was a good fit for each of the fiber groups, indicating that the drug release mechanism is controlled by diffusion of fingolimod from the PLGA fibers (Higuchi, 1963; Dash et al., 2010; Wu et al., 2020). Furthermore, the Korsmeyer–Peppas model was fit to the release data, as this model is used to describe drug release from swellable, polymeric systems like that of the PLGA fibers (Ritger and Peppas, 1987; Dash et al., 2010). This model was also found to be a good fit for the fingolimod release data, and the release exponent (*n*) for each fiber group indicated release *via* anomalous transport. Anomalous transport refers to a combination of Fickian diffusion and case II transport, indicating that the drug release is controlled by a combination of diffusion and swelling/erosion (Ritger and Peppas, 1987; Sujja-areevath et al., 1998; Siepmann and Peppas, 2001; Wu et al., 2020). These findings corroborate the speculations made in the previous paragraph that fingolimod release from the PLGA fibers is controlled by a combination of diffusion, swelling, and erosion.

Following material characterization, we tested the efficacy of the electrospun fiber scaffolds to elicit neurite extension from whole and dissociated DRG *in vitro*. We observed a significant increase in neurite number, extension, and branching when cultured on fingolimod-releasing fibers, in particular, on the 0.02% fingolimod-loaded fibers. Previous studies demonstrated the ability of fingolimod to increase BDNF expression in neurons (Deogracias et al., 2012; Doi et al., 2013; Anastasiadou and Knöll, 2016). BDNF increases neurite outgrowth, neurite branching, and the number of primary neurites (Lindsay, 1988; Deng et al., 1999; Guo et al., 2014; Kalil and Dent, 2014). Doi and colleagues showed that exposure of mouse primary cortical neurons to a one-time 0.1-nM dose of fingolimod increased the production of BDNF in the neurons (Doi et al., 2013). All fingolimod-releasing fibers were predicted to release fingolimod at concentrations ranging from 0.1 nM to 2.7 nM fingolimod in 1 ml of culture media per day for at least 4 days. Therefore, all drug-loaded fibers were predicted to release fingolimod at a daily dose that is known to effectively upregulate neuronal BDNF production *in vitro* for the entirety of the *in vitro* experiments. Furthermore, Anastasiadou and Knöll observed that the administration of a 50-nM dose of fingolimod significantly increased neurite outgrowth from adult mouse dissociated DRG neurons, and to our knowledge, this is the lowest dose of fingolimod previously assessed for its ability to increase neurite outgrowth from DRG neurons *in vitro* (Anastasiadou and Knöll, 2016). Thus, this study presents the unique finding that the sustained release of fingolimod from aligned electrospun fibers at concentrations lower than those previously investigated can stimulate whole and dissociated DRG neurite outgrowth.

Although fingolimod released from aligned electrospun fibers was effective for the 0.0004 and 0.02% fingolimod-loaded fiber groups, we did not observe a significant increase in whole DRG neurite extension when cultured on the 0.04% fingolimod-loaded fibers. Anastasiadou and Knöll found that fingolimod did not begin to limit neurite extension of cortical neurons until reaching concentrations of 100 nM, which is well above the concentration range predicted from an individual 0.04% fiber scaffold in 1 ml of culture media throughout the entirety of the *in vitro* experiments conducted (Anastasiadou and Knöll, 2016). Therefore, we speculate that the dose of fingolimod released from the 0.04% electrospun fibers is not high enough to limit neurite outgrowth from whole DRG. Wang et al. (2010) showed that crossing fibers can act as a barricade and impede neurite extension. Thus, a possible explanation for the decrease in neurite extension is the decreasing trend in fiber alignment observed with increasing concentrations of fingolimod in PLGA fibers. This indicates that the electrospinning parameters and the electrospinning solution composition may need to be adjusted when loading higher concentrations of fingolimod in order to maintain fiber alignment and support increased neurite outgrowth.

To investigate the effect of fingolimod-releasing fibers on Schwann cell phenotype, we first measured Schwann cell migration distance outward from the whole DRG body. This assessment is important when investigating the regenerative potential of a material for the PNS because the migration of Schwann cells can stimulate greater axon extension (Torigoe et al., 1996; Zhou et al., 2020). Schwann cell migration was significantly greater on all fingolimod-releasing fiber scaffolds compared with the control fibers, with the greatest increase observed on the 0.02% fingolimod-loaded fibers. Heinen and colleagues showed that a 100-nM dose of fingolimod *in vitro* increased the Schwann cell production of the transcription factor cJun (Heinen et al., 2015), which is associated with a pro-regenerative Schwann cell phenotype and enhanced migration capabilities (Huang et al., 2015). However, the amount of fingolimod released from an individual fingolimod-loaded fiber scaffold from all drug-loaded fiber groups was lower, both per day and cumulatively, than the 100-nM dose reported to modulate the Schwann cell gene expression of cJun (Heinen et al., 2015). Additionally, we did not observe a significant change in the Schwann cell mRNA expression levels of cJun following culture on the 0.02% fingolimod-loaded fibers. Heermann et al. (2011) showed that, if neurites extend outward from the ganglion body first, then Schwann cells will closely associate with the neurites and migrate outward along those extending neurites. The maximum Schwann cell migration distance was comparable to the whole DRG neurite extension length for each fiber group; thus, the enhanced Schwann cell migration from the DRG explants cultured on fingolimod-containing fibers may be a result of the Schwann cells migrating close to the neurites that extend out from the DRG body rather than the released fingolimod directly stimulating Schwann cell migration.

To further investigate Schwann cell phenotype, we looked at the mRNA expression levels of several regenerative and myelinating factors that fingolimod is known to modulate in addition to cJun (Heinen et al., 2015). We found that the 0.02% fingolimod-loaded fibers did not significantly affect the Schwann cell mRNA expression levels of the pro-regenerative factors investigated; however, they did significantly downregulate the Schwann cell mRNA expression levels of promyelinating factors. Köhne et al. (2012) showed that daily treatment of primary rat dissociated DRG (neuron/Schwann cell) cultures with a 100-nM dose of fingolimod significantly reduced the Schwann cell formation of myelin in culture and treatment with a 10-nM dose of fingolimod, although not significant, trended toward a decrease in myelin formation. Furthermore, Heinen et al. (2015) showed that the administration of a 100-nM dose of fingolimod significantly altered the Schwann cell expression of several pro-regenerative and promyelinating factors. This 100-nM dose is well above that which is being released from the aligned fingolimod-loaded fibers; thus, the presence of a small but significant decrease in the mRNA expression levels of the pro-myelinating factors tested indicates that the fingolimod-releasing fibers may begin to support changes in Schwann cells that are associated with phenotypic change but do not release fingolimod at a high-enough dose to fully promote the transition of Schwann cells to a pro-regenerative phenotype.

Although the increase in Schwann cell migration and the reduction in Schwann cell mRNA expression levels of promyelinating factors hinted that the 0.02% fingolimod-loaded fibers promote a regenerative Schwann cell phenotype, the lack of a significant change in the mRNA expression levels of pro-regenerative factors, most notably cJun, counteracts this idea. Chew et al. (2008) reported that aligned electrospun fibers induce a promyelinating phenotype in Schwann cells. Thus, the effects of the aligned electrospun fibers on Schwann cell phenotype may be enough to offset the effects from such a low dose of fingolimod, limiting changes in gene expression.

## Conclusion

In conclusion, fingolimod-loaded PLGA fibers extended the release of fingolimod hydrochloride for 28 days. To our knowledge, this is the longest duration of fingolimod release reported from electrospun fibers. Furthermore, this is the first study to investigate DRG neurite outgrowth and Schwann cell phenotype following culture on highly aligned, fingolimod-releasing electrospun fibers. Fingolimod release from the aligned PLGA fibers enhanced neurite outgrowth from the whole and dissociated DRG neurons as well as increased the Schwann cell migration and reduced the Schwann cell expression levels of promyelinating factors. Although the aligned fingolimod-releasing fibers were unable to shift the Schwann cells to a repair-supporting phenotype, these fibers increased neurite outgrowth from the whole and dissociated DRG neurons, indicating that they had a neurotrophic effect on the *in vitro* cultures. The regenerative properties of the fingolimod-releasing fibers observed *in vitro* show the potential of this material to enhance regeneration following peripheral nerve injury and support the reasoning for future testing of this material in an *in vivo* peripheral nerve injury model.

## Data Availability Statement

The datasets generated for this study can be found in the Mendeley Data Repository: https://data.mendeley.com/datasets/nchxcyvb6v/draft?a=83e3ac24-c804-4085-845a-1f9bba4c8560.

## Ethics Statement

All animal procedures were approved by the Institutional Animal Care and Use Committee (IACUC) at Rensselaer Polytechnic Institute (DRG isolation) or the University of Miami (Schwann cell isolation).

## Author Contributions

DP wrote the manuscript. DP, JF, and AD’A contributed to planning the study. DM fabricated the films. DP fabricated the electrospun fibers and characterized the fiber surface wettability. JB characterized the fiber morphology. DP, JB, and DZ conducted the fingolimod release assessment. DP and AD’A isolated, dissociated, and cultured the dorsal root ganglia (DRG). DP, AD’A, JB, and DM imaged and analyzed the whole DRG neurite extension, Schwann cell migration, and dissociated DRG neurite outgrowth. YP and AH isolated and purified the Schwann cells. DP and JF conducted the Schwann cell qPCR experiments. JF, AD’A, JB, DZ, MO, and RG edited the manuscript. All the authors approved the final version of the manuscript.

## Conflict of Interest

The authors declare that the research was conducted in the absence of any commercial or financial relationships that could be construed as a potential conflict of interest.

## Abbreviations

BDNF: brain-derived neurotrophic factor
BSA: bovine serum albumin
Cx32: connexin-32
DRG: dorsal root ganglia
GAP43: growth-associated protein 43
GAPDH: glyceraldehyde 3-phosphate dehydrogenase
HFP: 1,1,1,3,3,3-hexafluoro-2-propanol
HPLC: high-performance liquid chromatography
LCMS: liquid chromatography–mass spectrometry
MBP: myelin basic protein
mRNA: messenger ribonucleic acid
NCAM1: neural cell adhesion molecule 1
Oct6: octamer-binding factor 6
PBS: phosphate-buffered saline
PDGF-BB: platelet-derived growth factor-BB
PDMS: polydimethylsiloxane
PLGA: poly(lactic-co-glycolic acid)
PLLA: poly-L-lactic acid
PMP2: peripheral myelin protein 2
qPCR: quantitative polymerase chain reaction
S1P: sphingosine-1-phosphate
SEM: scanning electron microscopy.
