# Supplementary material for: Aligned Fingolimod-Releasing Electrospun Fibers Increase Dorsal Root Ganglia Neurite Extension and Decrease Schwann Cell Expression of Promyelinating Factors
Source: Front Bioeng Biotechnol. 2020 Aug 14;8:937. doi: 10.3389/fbioe.2020.00937 (PMC7456907; doi:10.3389/fbioe.2020.00937)
Supplement: Supplementary file 1 [file Table_1.DOCX]

Supplementary Material

# Materials Information

# Table S1. Materials and Product Information

| **Item** | **Company** | **Location** | **Product #** | **Lot #** |
| --- | --- | --- | --- | --- |
| 1,1,1,3,3,3-hexafluoro-2-propanol | Sigma | St. Louis, MO | 105228 | WXBL5954V |
| 4′,6-diamidino-2-phenylindole (DAPI) | Thermo Fisher | Waltham, MA | 62247 | -- |
| Alexa Fluor 488 donkey anti-mouse | Sigma | Waltham, MA | A21202 | 1915874 |
| Alexa Fluor 594 goat anti-rabbit secondary antibody | Sigma | Waltham, MA | A11037 | 2005936 |
| Autosampler vials kit | Thermo Fisher | Rockwood, TN | C5000-80W | 240412 |
| B-27 supplement | Gibco | Grand Island, NY | 17504-044 | 2004470 |
| BD 5 mL syringe Luer-Lok | Becton Dickinson and Company | Franklin Lakes, NJ | 309646 | 9148999 |
| Bovine serum albumin | Sigma | St. Louis, MO | A9647 | SLBT4366 |
| Collagenase | Sigma | St. Louis, MO | C9891 | -- |
| Dispase | Gibco | Japan | 17105-041 | 292972 |
| DMEM | Gibco | Grand Island, NY | 12800082 | 1949278 |
| Ethylene oxide | Anprolene | Haw River, NC | AN73 | 161331 |
| Fetal bovine serum | Gibco | Grand Island, NY | 10437028 | 2086961 |
| Fingolimod hydrochloride | LC Laboratories | Woburn, MA | F-4633 | FGD-109 |
| Fingolimod-D4 hydrochloride | Toronto Research Chemicals | Toronto, ON, Canada | F805002 | 6-ZCA-137-6 |
| Forskolin | Fisher Scientific | San Diego, CA | 50596449 | A01061 |
| Glass coverslips | Ted Pella | Redding, CA | 260377-1 | -- |
| Ham’s F12 nutrient mixture | Gibco | Gaithersburg, MD | 11765-054 | 1949928 |
| Hank’s Balanced Salt Solution | Gibco | Gaithersburg, MD | 14025134 | 1951077 |
| Heregulin | Peprotech | Rocky Hill, NJ | 50819211 | 0317316 |
| L-glutamine | Gibco | Grand Island, NY | 25030-081 | -- |
| Laminin | Gibco | Carlsbad, CA | 23017015 | 1963101, 2007488 |
| Mouse polyclonal RT-97 primary antibody | DSHB | Iowa City, Iowa | AB_528399 | Batch 9/28/17 |
| Neurobasal medium | Gibco | Grand Island, NY | 12348-017 | 2023035 |
| Nerve growth factor | Sigma | St. Louis, MO | N6009 | SLBV7658 |
| P2-P3 Sprague Dawley Rats | P2 Taconic Biosciences, P3 Envigo | -- | -- | -- |
| Paraformaldehyde | Electron Microscopy Sciences | Hatfield, PA | 15710 | 171207-06 |
| Penicillin-streptomycin | Gibco | Grand Island, NY | 15140-122 | 1999371 |
| PerfeCTa SYBR Green FastMix ROX | Quanta Biosciences | Gaithersburg, MD | 95073-012 | 66143731 |
| Phosphate buffered saline (PBS) | VWR | Solon, OH | E404 | 19B0356218 |
| Pituitary extract | Alfa Aesar | Haverhill, MA | J64417 (BT-215) | 215K18A |
| Poly(lactic-co-glycolic acid) | Lactel Absorbable Polymers | Birmingham, AL | B6010-4 | A16-108 |
| Poly-L-lysine | Sigma | St. Louis, MO | P4707 | RNBG8803 |
| Polypropylene inserts | Thermo Fisher | Langerwehe, Germany | C4010-630P | 8000018298 |
| Primer sets | Integrated DNA Technologies | Coralville, IA | -- | -- |
| qScript™ cDNA SuperMix | Quanta Biosciences | Gaithersburg, MD | 101414-106 | 24399 |
| Rabbit Complement | Bio-Rad | Hercules, CA | C12CC | -- |
| Rabbit polyclonal S100 primary antibody | Dako | Santa Clara, CA | Z0311 | 54637 |
| Thy-1 | Hybridoma | Old Town Manassas, VA | TIB-103 | -- |
| Triton X-100 | Sigma | St. Louis, MO | T8787 | MKBS6557V |
| TRIzol | Ambion | Waltham, MA | 15596018 | 175804 |
| TrypLE Express | Gibco | Grand Island, NY | 1260528 | -- |
| 2.5% Trypsin (10X) | Gibco | Grand Island, NY | 15090-046 | 2034610 |
| TWEEN-20 | Sigma | St. Louis, MO | P1379 | SLBT8195 |
| ZORBAX StableBond C18 column | Agilent | Santa Clara, CA | 860975-902 | -- |

# Table S2. Equipment and Software Information

| **Instrument/Software** | **Company** | **Location** |
| --- | --- | --- |
| 289 IX-81 Confocal Microscope | Olympus | Melville, NY |
| Adobe Photoshop CS2 | Adobe | San Jose, CA |
| Agilent 1200 Infinity Series high-performance liquid chromatography | Agilent | Palo Alto, CA |
| Expanded Plasma Cleaner | Harrick Plasma | Ithaca, NY |
| FIJI Version 2.0.0-rc-68 | National Institutes of Health | Bethesda, MD |
| Kruss DSA 100 | Kruss | Hamburg, Germany |
| Kruss ADVANCE software | Kruss | Hamburg, Germany |
| LC OpenLAB Software | Agilent | Santa Clara, CA |
| Metamorph Premier 7.7.3.0 | Molecular Devices | San Jose, CA |
| Minitab Software | Pennsylvania State University | State College, PA |
| Neurolucida Explorer software | MBF Bioscience | Williston, VT |
| STEPOne Real-Time PCR System | Applied Biosystems | Foster City, CA |
| Technics Hummer V Sputter Coater | Anatech USA | Hayward, CA |
| TSQ Quantum Ultra™ Triple Quadrupole Mass Spectrometer | Thermo | Bremen, Germany |
| Versa 3D Dual Beam Scanning Electron Microscope | FEI | Hillsboro, OR |
| Xcalibur® | Agilent | Santa Clara, CA |

# Additional Methods

## Dissociated DRG Purity Assessment

Confocal images of dissociated DRG cultures seeded onto glass coverslips were used to determine culture purity. Cells were cultured for 12 h then fixed and stained against S100 and RT-97 and counterstained for DAPI (see main text for more detail). Ten fields of view were captured per coverslip using a confocal microscope and two coverslips were imaged per replicate. The total number of neurons and other cells (primarily Schwann cells) was determined per image, and the percentage of neurons per image was calculated. The average percentage of neurons was determined per replicate, and the average of those averages was used to determine the purity of the culture. Purity was assessed following 3 separate dissociations (n = 3).

# Additional Results

## Dissociated DRG Purity Assessment

Dissociated DRG cultures contained 13.61 ± 1.09% neurons and the remainder consisted of other cells (primarily Schwann cells).

# Additional Figures


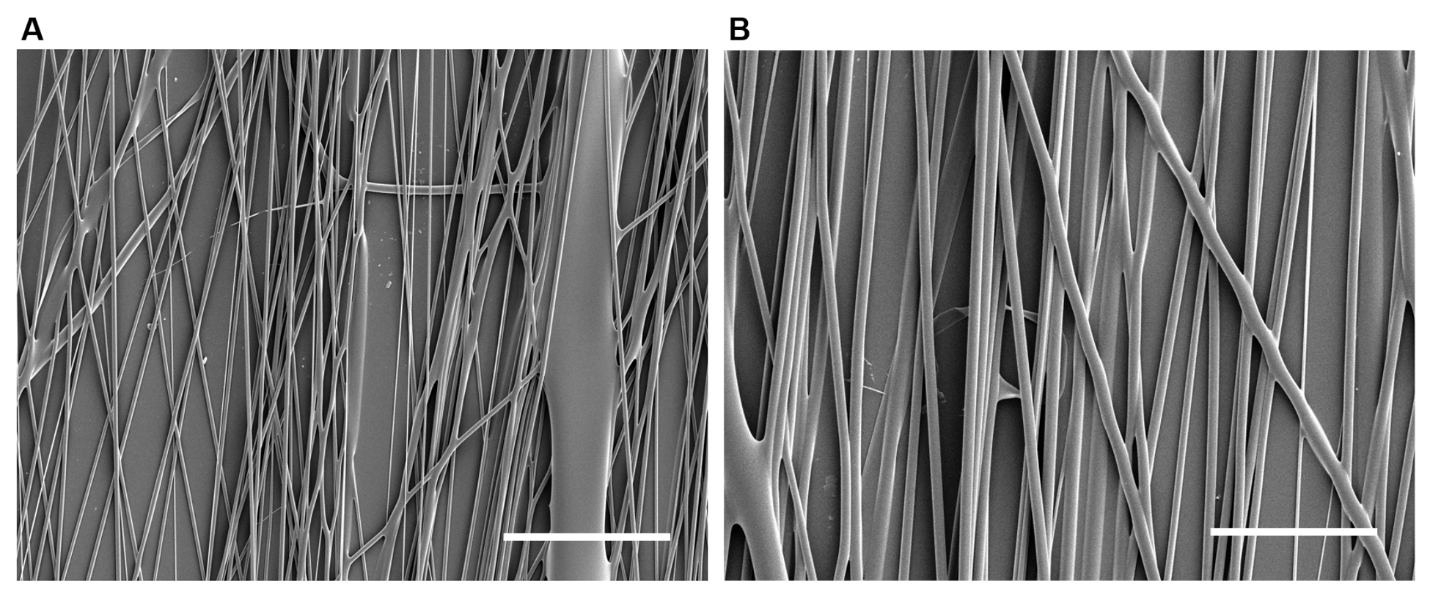


**Figure S1.** Incorporating 0.4% fingolimod into PLGA electrospun fibers caused poor fiber formation. SEM images of 0.4% fingolimod-loaded fibers taken at **(A)** 1000x (scale bar = 50 μm) and **(B)** 2500x (scale bar = 20 μm).


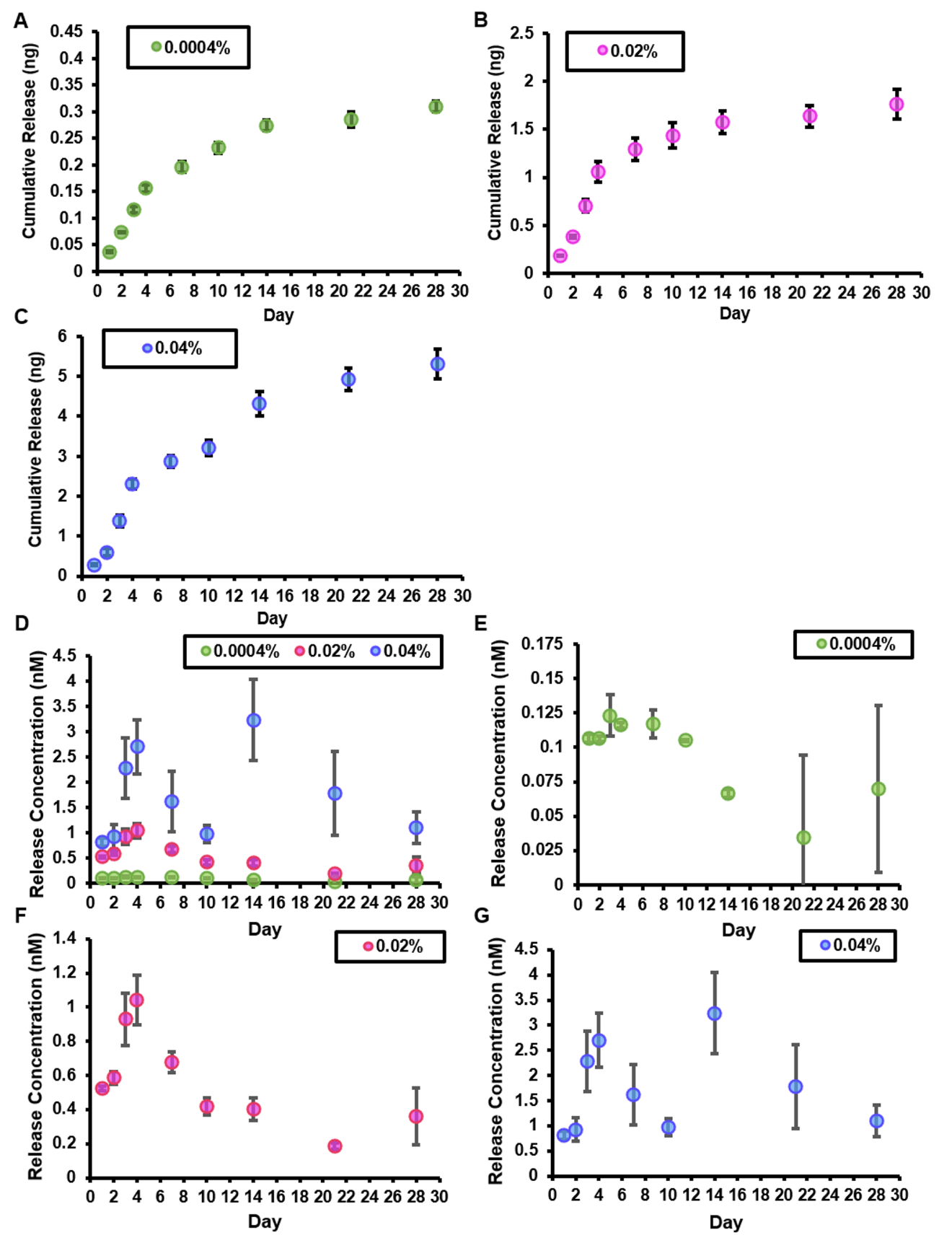


**Figure S2.** Fingolimod is continuously released from fingolimod-loaded electrospun PLGA fibers for at least 28 days. Predicted cumulative release of fingolimod from an individual **(A)** 0.0004%, **(B)** 0.02%, and **(C)** 0.04% fingolimod-loaded fiber scaffold represented by average mass (ng) ± standard deviation. Predicted concentration of fingolimod at each timepoint (not cumulative) when released into 1 mL of deionized water from **(D)** all, **(E)** 0.0004%, **(F)** 0.02%, and **(G)** 0.04% fingolimod-loaded fiber groups represented by average concentration (nM) ± standard deviation.


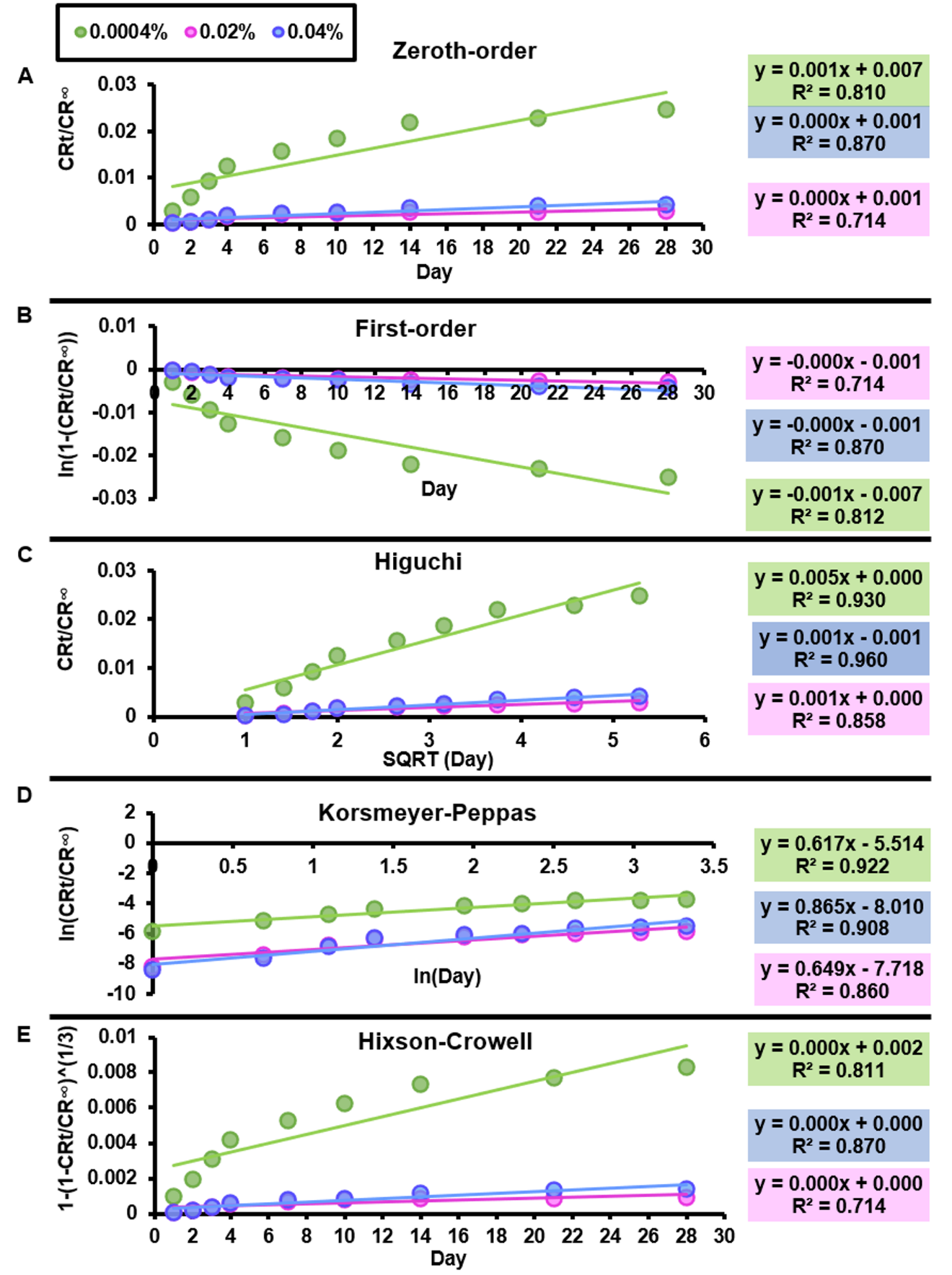


**Figure S3.** Kinetic models fit to *in vitro* fingolimod release data from 0.0004% (green), 0.02% (pink), and 0.04% (blue) fingolimod-releasing PLGA fibers. **(A)** Zero-order, **(B)** First-order, **(C)** Higuchi, **(D)**  Korsmeyer-Peppas, and **(E)** Hixson-Crowell kinetic models fit to the fingolimod cumulative release data.


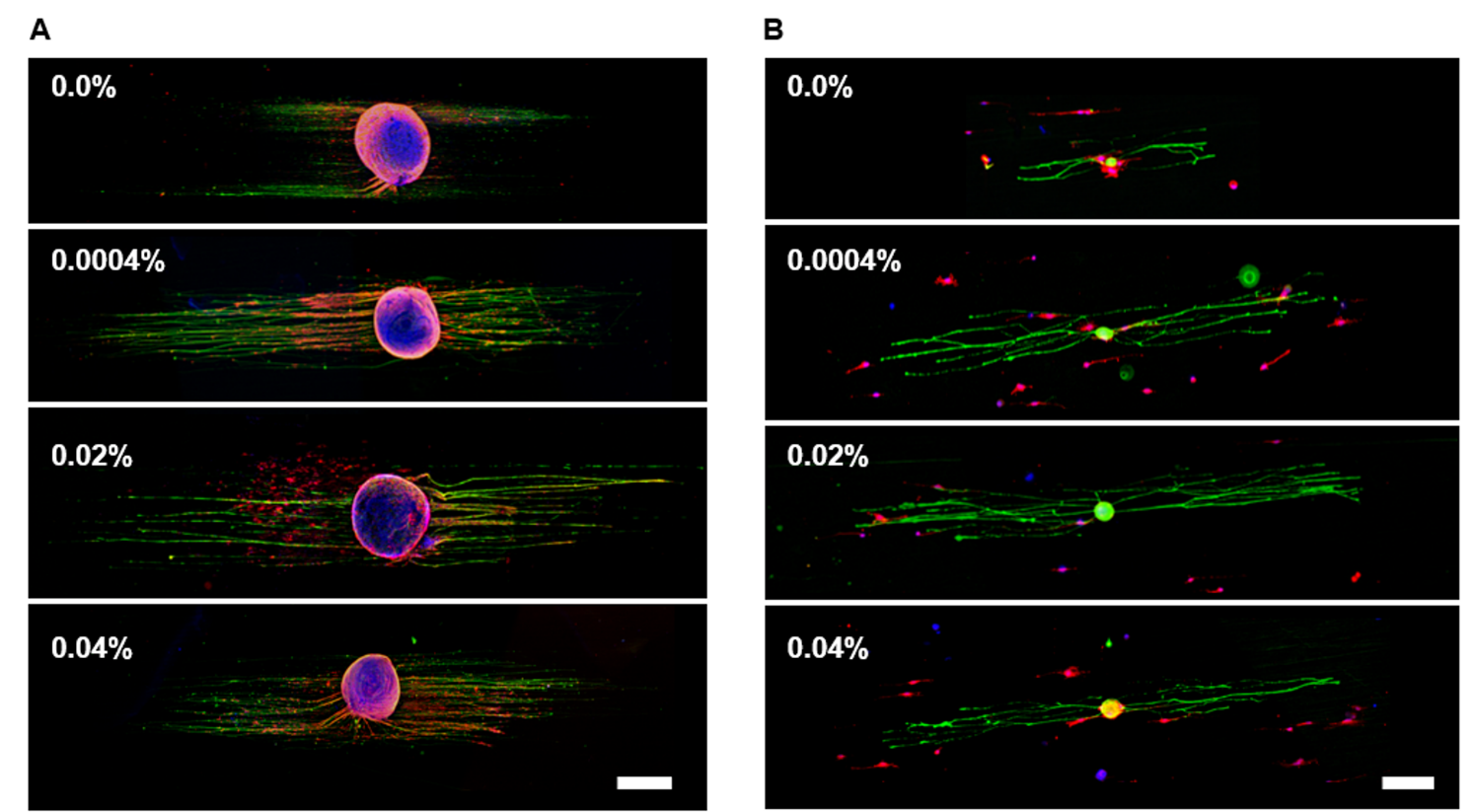


**Figure S4.** Fluorescent images of whole DRG explants and dissociated DRG cultures captured via confocal microscopy. Confocal images of **(A)** whole DRG cultured for 4 days (scale bar = 500 μm) and **(B)** individual DRG neurons cultured for 12 h (scale bar = 100 μm). Neurons were stained against neurofilament (green), Schwann cells were stained against S100 (red), and nuclei were counterstained with DAPI (blue).


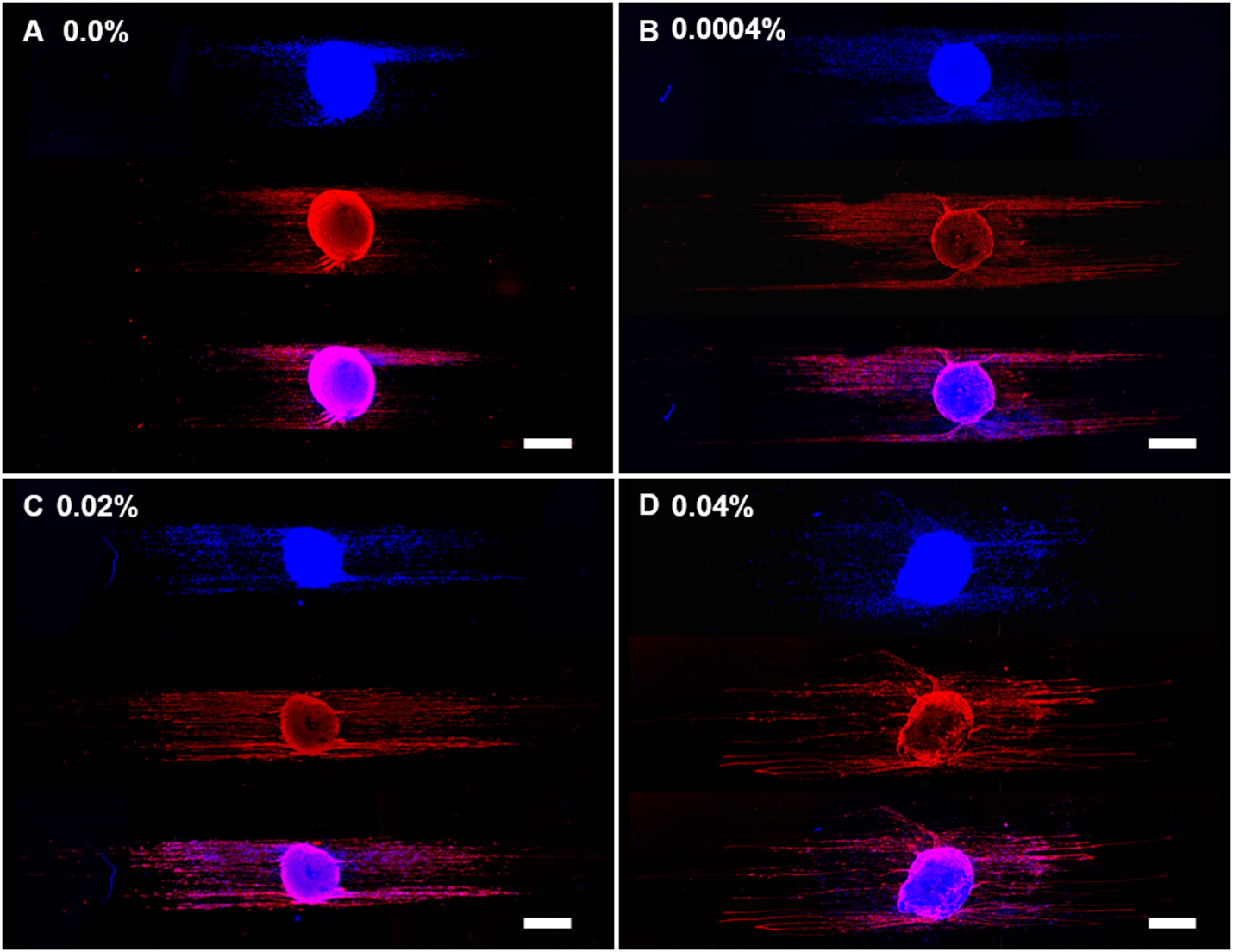


**Figure S5.** Fluorescent images of whole DRG explants captured via confocal microscopy. Whole DRG cultured for 4 days on **(A)** 0.0%, **(B)** 0.0004%, **(C)** 0.02%, and **(D)** 0.04% fingolimod-loaded fibers, counterstained with DAPI (blue), and stained against S100 (red). For each DRG (from top to bottom) the image shows the DAPI nuclear stain, Schwann cells stained against S100, and an overlay of both channels (scale bar = 500 µm).


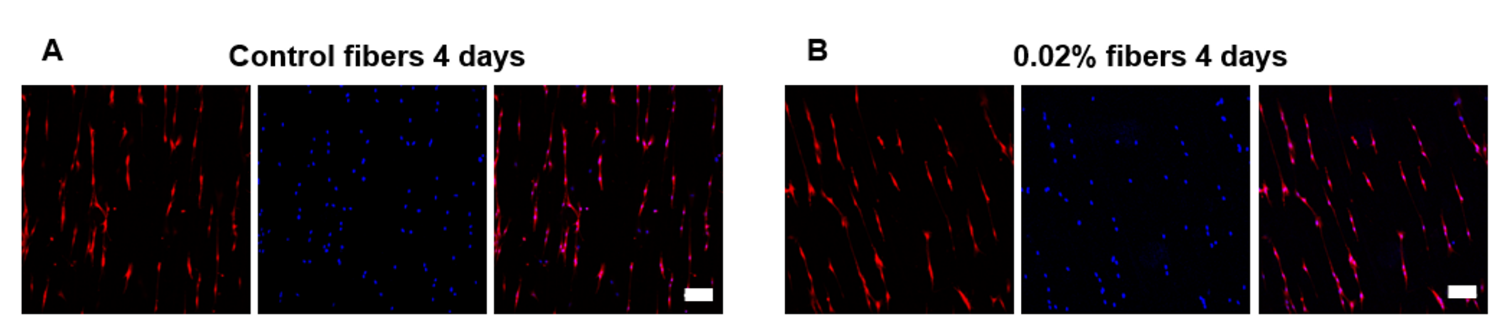


**Figure S6.** Fluorescent images of purified Schwann cells captured via confocal microscopy. Schwann cells cultured for 4 days on **(A)** 0.0% control fibers and **(B)** 0.02% fingolimod-loaded fibers. From left to right images show Schwann cells stained against S100 (red), counterstained with DAPI (blue), and an overlay of both channels (scale bar = 100 µm).





**Figure S7.** Preliminary assessment of Schwann cell mRNA expression levels at the 1-day time point via qPCR. RNA fold change in Schwann cells cultured for 1 day on control PLGA fibers and exposed to no fingolimod or a 100-nM bolus of fingolimod at day zero. A single replicate was used for the preliminary assessment of RNA fold change (n = 1).





**Figure S8.** Preliminary assessment of Schwann cell mRNA expression levels at the 4-day time-point via qPCR. RNA fold change in Schwann cells cultured for 4 days on control PLGA fibers and exposed to no fingolimod or a 100-nM bolus of fingolimod at day zero. A single replicate was used for the preliminary assessment of RNA fold change (n = 1).

# Statistical Information

## Additional Statistical Information for Fiber Morphology Characterization

We conducted a Moods Median test to assess changes in fiber alignment, a Welch’s ANOVA with a post hoc Games Howell test to assess changes in fiber diameter, and a one-way ANOVA with post hoc Dunnett’s test to assess changes in percent fiber coverage (Table S3–S8).

**Table S3.** Mood’s Median test used to assess changes in fiber alignment (α = 0.05).

| **DF** | **Chi-Square** | **P-Value** |
| --- | --- | --- |
| 3 | 0.133 | 0.988 |

**Table S4.** 95% Confidence intervals for 0.0%, 0.0004%, 0.02%, and 0.04% percent fiber alignment. The deviation from the mean fiber angle was assessed, so the mean of each group is zero.

| **Condition** | **N** | **Mean** | **StDev** | **95% CI** |
| --- | --- | --- | --- | --- |
| 0.0% | 300 | 0.000 | 3.917 | (-0.445, 0.445) |
| 0.0004% | 300 | 0.000 | 3.349 | (-0.381, 0.381) |
| 0.02% | 300 | 0.000 | 4.617 | (-0.525, 0.525) |
| 0.04% | 300 | 0.000 | 5.546 | (-0.630, 0.630) |

**Table S5.** Welch’s ANOVA used to determine changes in fiber diameter (α = 0.05).

| **DF** | **F-Value** | **P-Value** |
| --- | --- | --- |
| 3 | 1.721 | 0.161 |

**Table S6.** 95% Confidence intervals for 0.0%, 0.0004%, 0.02%, and 0.04% fiber diameter.

| **Condition** | **N** | **Mean** | **StDev** | **95% CI** |
| --- | --- | --- | --- | --- |
| 0.0% | 300 | 1.004 | 0.212 | (0.980, 1.028) |
| 0.0004% | 300 | 1.023 | 0.219 | (0.998, 1.048) |
| 0.02% | 300 | 0.988 | 0.203 | (0.965, 1.011) |
| 0.04% | 300 | 1.020 | 0.220 | (0.995, 1.045) |

**Table S7.** One-way ANOVA used to assess changes in percent fiber coverage (α = 0.05).

| **DF** | **F-Value** | **P-Value** |
| --- | --- | --- |
| 3 | 2.589 | 0.070 |

**Table S8.** 95% Confidence intervals for 0.0%, 0.0004%, 0.02%, and 0.04% percent fiber coverage.

| **Condition** | | **N** | **Mean** | | | **StDev** | **95% CI** | |  |
| --- | --- | --- | --- | --- | --- | --- | --- | --- | --- |
| 0.0% | | 9 | 78.184 | | | 3.469 | (75.753, 80.616) | |  |
| 0.0004% | | 9 | 78.260 | | | 4.086 | (75.828, 80.691) | |  |
| 0.02% | | 9 | 74.712 | | | 2.657 | (72.281, 77.144) | |  |
| 0.04% | | 9 | 75.102 | | | 3.939 | (72.671, 77.534) | |  |
|  |  | | |  |  | | |  | |

## Additional Statistical Information for Fiber Contact Angle

We conducted Welch’s ANOVA with post hoc Games-Howell test and 95% confidence interval to determine changes in static water contact angle on 0.0%, 0.0004%, 0.02%, and 0.04% fiber groups (Table S9–S11). Although the *p*-value shows significance, the statistical difference was between 0.02% and 0.04% fiber groups. None of the fingolimod-loaded fiber groups were statistically different from the 0.0% control fiber group.

**Table S9.** Welch’s ANOVA used to determine changes in the static water contact angle (α = 0.05).

| **DF** | **F-Value** | **P-Value** |
| --- | --- | --- |
| 3 | 5.250 | 0.004 |

**Table S10.** 95% Confidence intervals for 0.0%, 0.0004%, 0.02%, and 0.04% fiber scaffold static water contact angle.

| **Condition** | **N** | **Mean** | **StDev** | **95% CI** |
| --- | --- | --- | --- | --- |
| 0.0% | 22 | 105.020 | 6.579 | (102.106, 107.940) |
| 0.0004% | 18 | 107.900 | 3.677 | (106.072, 109.728) |
| 0.02% | 26 | 108.319 | 4.045 | (106.685, 109.953) |
| 0.04% | 18 | 105.356 | 1.672 | (104.524, 106.187) |

**Table S11.** Groups determined via the Games-Howell Method and the 95% confidence. The same letter indicates the group is the same.

| **Condition** | **Grouping** | |
| --- | --- | --- |
| 0.0% | A | B |
| 0.0004% | A | B |
| 0.02% | A |  |
| 0.04% |  | B |

## Additional Statistical Information for Whole DRG Adhesion

We utilized a binary logistic regression with link function: Logit to characterize changes in whole DRG adhesion to 0.0%, 0.0004%, 0.02% and 0.04% fiber scaffolds (Table S12–S13).

**Table S12.** Adhesion Statistics for Whole DRG cultured on 0.0%, 0.0004%, 0.02%, and 0.04% fiber scaffolds (α = 0.05).

| **Source** | **DF** | **Chi-Square** | **P-Value** |
| --- | --- | --- | --- |
| Regression | 3 | 2.723 | 0.436 |
| Electrospun Fibers | 3 | 2.723 | 0.436 |

**Table S13.** Odds ratios and 95% Confidence intervals for whole DRG adhesion on 0.0% (0), 0.0004% (1), 0.02% (2), and 0.04% (3).

| **Level A** | **Level B** | **Odds Ratio** | **95% CI** |
| --- | --- | --- | --- |
| 1 | 0 | 1.855 | (0.667, 5.154) |
| 2 | 0 | 2.700 | (0.691, 10.547) |
| 3 | 0 | 1.333 | (0.430, 4.134) |
| 2 | 1 | 1.456 | (0.359, 5.909) |
| 3 | 1 | 0.719 | (0.222, 2.334) |
| 3 | 2 | 0.494 | (0.112, 2.175) |

## Additional Statistical Information for Whole DRG Neurite Extension

We conducted a one-way ANOVA with post hoc Dunnett’s test to assess changes in neurite outgrowth following whole DRG cultured on 0.0%, 0.0004%, 0.02%, and 0.04% electrospun fibers (Tables S14–S16).

**Table S14.** One-way ANOVA used to determine changes in whole DRG neurite outgrowth (α = 0.05).

| **DF** | **F-Value** | **P-Value** |
| --- | --- | --- |
| 3 | 11.941 | 0.000 |

**Table S15.** 95% Confidence intervals for 0.0%, 0.0004%, 0.02%, and 0.04% whole DRG neurite extension.

| **Condition** | **N** | **Mean** | **StDev** | **95% CI** |
| --- | --- | --- | --- | --- |
| 0.0% | 30 | 1639.465 | 523.468 | (1442.719, 1836.211) |
| 0.0004% | 29 | 2100.822 | 530.123 | (1900.713, 2300.931) |
| 0.02% | 14 | 2570.417 | 514.793 | (2282.411, 2858.424) |
| 0.04% | 20 | 1663.883 | 603.387 | (1422.919, 1904.846) |

**Table S16.** Groups determined via the Dunnett’s Method and the 95% confidence. The same letter indicates the group is the same.

| **Condition** | **Grouping** |
| --- | --- |
| 0.0% | A |
| 0.0004% |  |
| 0.02% |  |
| 0.04% | A |

## Additional Statistical Information for Individual Neuron Neurite Morphology Analysis

We conducted a Welch’s ANOVA and post hoc Games-Howell test to assess changes in total neurite length, longest neurite, and number of branch points and a one-way ANOVA and post hoc Dunnett’s test to assess changes in the number of primary neurites (Table S17-S28).

**Table S17.** Welch’s ANOVA used to determine changes in individual DRG neuron total neurite length (α = 0.05).

| **DF** | **F-Value** | **P-Value** |
| --- | --- | --- |
| 3 | 15.228 | 0.000 |

**Table S18.** 95% Confidence intervals for 0.0%, 0.0004%, 0.02%, and 0.04% individual DRG neuron total neurite length.

| **Condition** | **N** | **Mean** | **StDev** | **95% CI** |
| --- | --- | --- | --- | --- |
| 0.0% | 30 | 1362.147 | 1123.031 | (942.800, 1781.493) |
| 0.0004% | 33 | 2393.355 | 1078.423 | (2010.963, 2775.747) |
| 0.02% | 31 | 3476.429 | 1594.031 | (2891.734, 4061.124) |
| 0.04% | 32 | 2965.800 | 1224.278 | (2524.401, 3407.199) |

**Table S19.** Groups determined via the Games-Howell Method and the 95% confidence. The same letter indicates the group is the same.

| **Condition** | **Grouping** | | |
| --- | --- | --- | --- |
| 0.0% | A |  |  |
| 0.0004% |  | B |  |
| 0.02% |  |  | C |
| 0.04% |  | B | C |

**Table S20.** Welch’s ANOVA used to determine changes in individual DRG neuron longest neurite length (α = 0.05).

| **DF** | **F-Value** | **P-Value** |
| --- | --- | --- |
| 3 | 14.957 | 0.000 |

**Table S21.** 95% Confidence intervals for 0.0%, 0.0004%, 0.02%, and 0.04% individual DRG neuron longest neurite length.

| **Condition** | **N** | **Mean** | **StDev** | **95% CI** |
| --- | --- | --- | --- | --- |
| 0.0% | 30 | 268.080 | 100.244 | (230.648, 305.512) |
| 0.0004% | 33 | 414.548 | 98.436 | (379.645, 449.452) |
| 0.02% | 31 | 420.997 | 100.523 | (384.125, 457.869) |
| 0.04% | 32 | 372.462 | 89.244 | (340.287, 404.638) |

**Table S22.** Groups determined via the Games-Howell Method and the 95% confidence. The same letter indicates the group is the same.

| **Condition** | **Grouping** | |
| --- | --- | --- |
| 0.0% | A |  |
| 0.0004% |  | B |
| 0.02% |  | B |
| 0.04% |  | B |

**Table S23.** Welch’s ANOVA used to determine changes in individual DRG neuron number of branch points (α = 0.05).

| **DF** | **F-Value** | **P-Value** |
| --- | --- | --- |
| 3 | 10.951 | 0.000 |

**Table S24.** 95% Confidence intervals for 0.0%, 0.0004%, 0.02%, and 0.04% individual DRG neuron number of branch points.

| **Condition** | **N** | **Mean** | **StDev** | **95% CI** |
| --- | --- | --- | --- | --- |
| 0.0% | 30 | 4.667 | 4.773 | (2.884, 6.449) |
| 0.0004% | 33 | 7.606 | 5.788 | (5.554, 9.658) |
| 0.02% | 31 | 11.645 | 6.964 | (9.091, 14.200) |
| 0.04% | 32 | 11.625 | 6.399 | (9.318, 13.932) |

**Table S25.** Groups determined via the Games-Howell Method and the 95% confidence. The same letter indicates the group is the same.

| **Condition** | **Grouping** | | |
| --- | --- | --- | --- |
| 0.0% | A |  |  |
| 0.0004% | A | B |  |
| 0.02% |  | B | C |
| 0.04% |  |  | C |

**Table S26.** One-way ANOVA used to determine changes in individual DRG neuron number of primary neurites (α = 0.05).

| **DF** | **F-Value** | **P-Value** |
| --- | --- | --- |
| 3 | 5.120 | 0.002 |

**Table S27.** 95% Confidence intervals for 0.0%, 0.0004%, 0.02%, and 0.04% individual DRG neuron number of primary neurites.

| **Condition** | **N** | **Mean** | **StDev** | **95% CI** |
| --- | --- | --- | --- | --- |
| 0.0% | 30 | 3.667 | 1.709 | (3.079, 4.254) |
| 0.0004% | 33 | 3.818 | 1.357 | (3.258, 4.378) |
| 0.02% | 31 | 5.129 | 1.784 | (4.551, 5.707) |
| 0.04% | 32 | 4.344 | 1.638 | (3.775, 4.913) |

**Table S28.** Groups determined via the Dunnett’s Method and the 95% confidence. The same letter indicates the group is the same.

| **Condition** | **Grouping** | |
| --- | --- | --- |
| 0.0% | A |  |
| 0.0004% | A |  |
| 0.02% |  |  |
| 0.04% | A |  |

## Additional Statistical Information for Schwann Cell Migration from the DRG Body

We used a one-way ANOVA and post hoc Dunnett’s test to determine changes in Schwann cell migration outward from the whole DRG body (Table S29–S31)

**Table S29.** One-way ANOVA used to determine changes in Schwann cell migration distance (α = 0.05).

| **DF** | **F-Value** | **P-Value** |
| --- | --- | --- |
| 3 | 11.649 | 0.000 |

**Table S30.** 95% Confidence intervals for 0.0%, 0.0004%, 0.02%, and 0.04% Schwann cell migration distance.

| **Condition** | **N** | **Mean** | **StDev** | **95% CI** |
| --- | --- | --- | --- | --- |
| 0.0% | 30 | 1761.104 | 461.361 | (1548.550, 1973.658) |
| 0.0004% | 28 | 2379.976 | 528.396 | (2159.962, 2599.991) |
| 0.02% | 14 | 2793.031 | 766.751 | (2481.884, 3104.179) |
| 0.04% | 20 | 2359.148 | 682.369 | (2098.823, 2619.472) |

**Table S31.** Groups determined via the Dunnett’s Method and the 95% confidence. The same letter indicates the group is the same.

| **Condition** | **Grouping** |
| --- | --- |
| 0.0% | A |
| 0.0004% |  |
| 0.02% |  |
| 0.04% |  |

## Additional statistical information for Schwann cell gene expression analysis

We used a general linear regression to assess changes in Schwann cell gene expression following culture on either 0.0% or 0.02% electrospun fibers (Table S32-S41).

**Table S32.** General linear regression used to determine changes in Schwann cell expression of BDNF (α = 0.05).

| **Gene** | **DF** | **T-Value** | **F-Value** | **P-Value** |
| --- | --- | --- | --- | --- |
| BDNF | 1 | -0.456 | 0.208 | 0.672 |

**Table S33.** General linear regression used to determine changes in Schwann cell expression of cJun (α = 0.05).

| **Gene** | **DF** | **T-Value** | **F-Value** | **P-Value** |
| --- | --- | --- | --- | --- |
| cJun | 1 | 1.627 | 2.646 | 0.179 |

**Table S34.** General linear regression used to determine changes in Schwann cell expression of GAP43 (α = 0.05).

| **Gene** | **DF** | **T-Value** | **F-Value** | **P-Value** |
| --- | --- | --- | --- | --- |
| GAP43 | 1 | -2.466 | 6.080 | 0.069 |

**Table S35.** General linear regression used to determine changes in Schwann cell expression of NCAM1 (α = 0.05).

| **Gene** | **DF** | **T-Value** | **F-Value** | **P-Value** |
| --- | --- | --- | --- | --- |
| NCAM1 | 1 | 1.116 | 1.246 | 0.327 |

**Table S36.** General linear regression used to determine changes in Schwann cell expression of PDGF-BB (α = 0.05).

| **Gene** | **DF** | **T-Value** | **F-Value** | **P-Value** |
| --- | --- | --- | --- | --- |
| PDGF-BB | 1 | -1.484 | 2.202 | 0.212 |

**Table S37.** General linear regression used to determine changes in Schwann cell expression of Cx32 (α = 0.05).

| **Gene** | **DF** | **T-Value** | **F-Value** | **P-Value** |
| --- | --- | --- | --- | --- |
| Cx32 | 1 | 1.930 | 3.725 | 0.126 |

**Table S38.** General linear regression used to determine changes in Schwann cell expression of Krox20 (α = 0.05).

| **Gene** | **DF** | **T-Value** | **F-Value** | **P-Value** |
| --- | --- | --- | --- | --- |
| Krox20 | 1 | 2.863 | 8.199 | 0.046 |

**Table S39.** General linear regression used to determine changes in Schwann cell expression of MBP (α = 0.05).

| **Gene** | **DF** | **T-Value** | **F-Value** | **P-Value** |
| --- | --- | --- | --- | --- |
| MBP | 1 | -0.243 | 0.059 | 0.820 |

**Table S40.** General linear regression used to determine changes in Schwann cell expression of Oct6 (α = 0.05).

| **Gene** | **DF** | **T-Value** | **F-Value** | **P-Value** |
| --- | --- | --- | --- | --- |
| Oct6 | 1 | 4.493 | 20.186 | 0.011 |

**Table S41.** General linear regression used to determine changes in Schwann cell expression of PMP2 (α = 0.05).

| **Gene** | **DF** | **T-Value** | **F-Value** | **P-Value** |
| --- | --- | --- | --- | --- |
| PMP2 | 1 | 3.843 | 14.768 | 0.018 |
